# Transcriptional Profile of the Developing Subthalamic Nucleus

**DOI:** 10.1523/ENEURO.0193-22.2022

**Published:** 2022-10-17

**Authors:** Ema Bokulić, Tila Medenica, Goran Sedmak

**Affiliations:** 1 Croatian Institute for Brain Research, University of Zagreb School of Medicine, Zagreb, 10000, Croatia; 2 Center of Excellence for Basic, Clinical and Translational Neuroscience, University of Zagreb School of Medicine, Zagreb, 10000, Croatia

**Keywords:** basal ganglia, hypothalamus, prosomeric model, subthalamic nucleus, transcription factors

## Abstract

The subthalamic nucleus (STN) is a small, excitatory nucleus that regulates the output of basal ganglia motor circuits. The functions of the STN and its role in the pathophysiology of Parkinson’s disease are now well established. However, some basic characteristics like the developmental origin and molecular phenotype of neuronal subpopulations are still being debated. The classical model of forebrain development attributed the origin of STN within the diencephalon. Recent studies of gene expression patterns exposed shortcomings of the classical model. To accommodate these findings, the prosomeric model was developed. In this concept, STN develops within the hypothalamic primordium, which is no longer a part of the diencephalic primordium. This concept is further supported by the expression patterns of many transcription factors. It is interesting to note that many transcription factors involved in the development of the STN are also involved in the pathogenesis of neurodevelopmental disorders. Thus, the study of neurodevelopmental disorders could provide us with valuable information on the roles of these transcription factors in the development and maintenance of STN phenotype. In this review, we summarize historical theories about the developmental origin of the STN and interpret the gene expression data within the prosomeric conceptual framework. Finally, we discuss the importance of neurodevelopmental disorders for the development of the STN and its potential role in the pathophysiology of neurodevelopmental disorders.

## Significance Statement

The subthalamic nucleus is functionally a part of the basal ganglia circuitry, but the accumulated evidence from analyzing gene expression patterns and lineage-tracing experiments point to a hypothalamic developmental origin. The expression of morphogens and transcription factors in the STN corroborates the hypothalamic origin. Interestingly, some of the genes expressed in the STN are relevant for human brain development as they are involved in the pathophysiology of neurodevelopmental disorders (NDDs). This review could serve as a guidepost for future research on the neuronal phenotypes of human STN and their roles in NDDs.

## Introduction

The subthalamic nucleus (STN) is a small, lens-shaped structure with excitatory efferents to the globus pallidus pars interna (GPi) and globus pallidus pars externa (GPe) and substantia nigra pars reticulata (SNr; Emmi et al., 2020). In humans, the STN is located ventral to the zona incerta (ZI) and the H2 field of Forel, dorsolateral to the SN and dorsal to capsula interna. The anterior medial border consists of lateral hypothalamic area, and the posterior medial border is the red nucleus (Hamani et al., 2004; Mai et al., 2004; Emmi et al., 2020). Currently, the STN has a prominent role in the treatment of movement disorders (Kumar et al., 1998; Hamani et al., 2005; Benabid et al., 2009). Therefore, the role of the STN in the basal ganglia circuitry and in the pathophysiology of Parkinson’s disease (PD) is well researched (for review, see Parent and Hazrati, 1995; Joel and Weiner, 1997; Hamani et al., 2004; Temel et al., 2005; Bevan et al., 2006). Although the cellular composition of the STN and its neuronal morphology have been studied in different species [e.g., rat (Kita et al., 1983; Afsharpour, 1985); guinea pig (Robak et al., 2000); Göttingen minipig (Larsen et al., 2004); cat (Iwahori, 1978; Romansky and Usunoff, 1985, 1987); monkey (Rafols and Fox, 1976; Sato et al., 2000); and human (Yelnik and Percheron, 1979; Hardman et al., 2002; Lévesque and Parent, 2005)], the role of various genes in generation, specification, and maintenance of STN neuronal populations is still largely underexplored.

Transcription factors (TFs) regulate gene expression, control developmental patterning, neuronal specification, migration, and maturation. While the role of TFs has been extensively studied in the developing cerebral cortex (for review, see Molyneaux et al., 2007; Popovitchenko and Rasin, 2017; Molnár et al., 2019), it has been poorly studied in subcortical structures. Structures like the thalamus and hypothalamus have been particularly tough to study because of their heterogeneous neuronal populations and a lack of cell type-specific markers (Blackshaw et al., 2010). However, studies of gene expression have slowly begun to elucidate transcriptional codes that specify distinct cell types in these forebrain areas (Shimogori et al., 2010; Suzuki-Hirano et al., 2011; Moffitt et al., 2018; Guo and Li, 2019; Mickelsen et al., 2019, 2020; Romanov et al., 2020; Wen et al., 2020; Zhang et al., 2021). In line with these advancements, a combinatorial transcriptional code defining the STN is emerging.

The developmental origin of STN is still highly debated, and the lack of consensus on the definitions of hypothalamus and diencephalon has hindered the research on the differentiation and specification of STNs. Historically, STN has been described as a diencephalic structure and a part of the subthalamus, which was considered a separate part of the diencephalon (Kuhlenbeck, 1954; Reinoso-Suarez, 1966; Richter, 1966; Müller and O’Rahilly, 1988). New studies describing gene expression patterns in the developing forebrain have challenged the historical division of the forebrain, and with that, the place of the STN within it. Therefore, the summary of transcriptional code defining the STN could shed light on this debate. However, an extensive search of literature did not provide any study that systematically summarizes the data on TF expression in STN, with the exception of one review that was not focused solely on TFs (Philips et al., 2005). The aim of this review is to clarify the meaning of these new data for the position of the STN, to provide an overview of transcription factors involved in STN development, and to use them to discuss the validity of some developmental theories. Finally, some of the discussed TFs are implicated in the pathogenesis of several neurodevelopmental disorders (NDDs), so we will try to link the functional roles of the STN with the symptomatology present in these disorders.

## Historical Perspectives—The Place of the Subthalamic Nucleus within Columnar and Prosomeric Models

Historically, the STN has been described as a diencephalic structure. In pioneering studies by Herrick (1910) of the amphibian brain, he proposed the columnar model of diencephalic development (based on anatomic landmarks), dividing the diencephalic primordium into the four horizontal/longitudinal subdivisions: epithalamus, dorsal thalamus, ventral thalamus, and hypothalamus. This model was an updated version of the original model of diencephalic development by His (1893a), who divided the diencephalon into epithalamus, thalamus, and hypothalamus. For years, the columnar model has been widely used as a basis to describe the development of the diencephalon with some modifications. Many authors interpreting the development of STN within the columnar model have placed the STN in the additional fifth diencephalic column, the subthalamus (Kuhlenbeck, 1954; Reinoso-Suarez, 1966; Richter, 1966; Müller and O’Rahilly, 1988). Besides the STN, the subthalamic column gave rise to the thalamic reticular nucleus, GP, and ZI (Reinoso-Suarez, 1966; Richter, 1966; Müller and O’Rahilly, 1988). However, the columnar model was challenged by neuroembryological studies of Bergquist and Källěn (1954, 1955), who found that the embryonic brain of all major vertebrates has transverse and longitudinal zones of intense proliferation that make up a grid-like pattern (Nieuwenhuys, 2017). Interestingly, these transverse zones of high mitotic activity have already been described in the late 19th/early 20th century by Von Baer (1828), Orr (1887), and Von Kupffer (1906), who acknowledged the segmental nature of the embryonic brain. Orr (1887) called these zones neuromeres or neural segments. Although the neuromeric models were developed before the columnar model was proposed, authors like Herrick (1910) and Kuhlenbeck (1954) disregarded neuromeres as transient structures that disappear during the course of embryonic development (Puelles, 2021). Another discrepancy between the early neuroembryological observations and the columnar model concerns the question of the forebrain axis. Columnar authors neglected the cephalic flexure and simply assumed that the straight forebrain axis ends in the telencephalon (Herrick, 1910; Kuhlenbeck, 1954; Puelles et al., 2012). Finally, the columnar model prevailed as the neuromeric models had several problems that could not be resolved without modern techniques of experimental embryology. For example, the neuromeric pattern in the embryonic rhombencephalon is easily distinguishable, whereas segmental organization of the prosencephalon is not so conspicuous. The proliferative zones (proneuromeres, neuromeres, and transverse bulges) of Bergquist and Källěn (1954, 1955) were thought to appear and disappear sequentially, making it difficult to establish boundaries between them (Nieuwenhuys, 2017; Puelles, 2021). Another problem was the number of forebrain neuromeres and the position of sulcus limitans, which directly influences our understanding of the position of motor and sensory nuclei in the prosencephalon (Keyser, 1973; Gribnau and Geijsberts, 1985).

**Figure 1.**
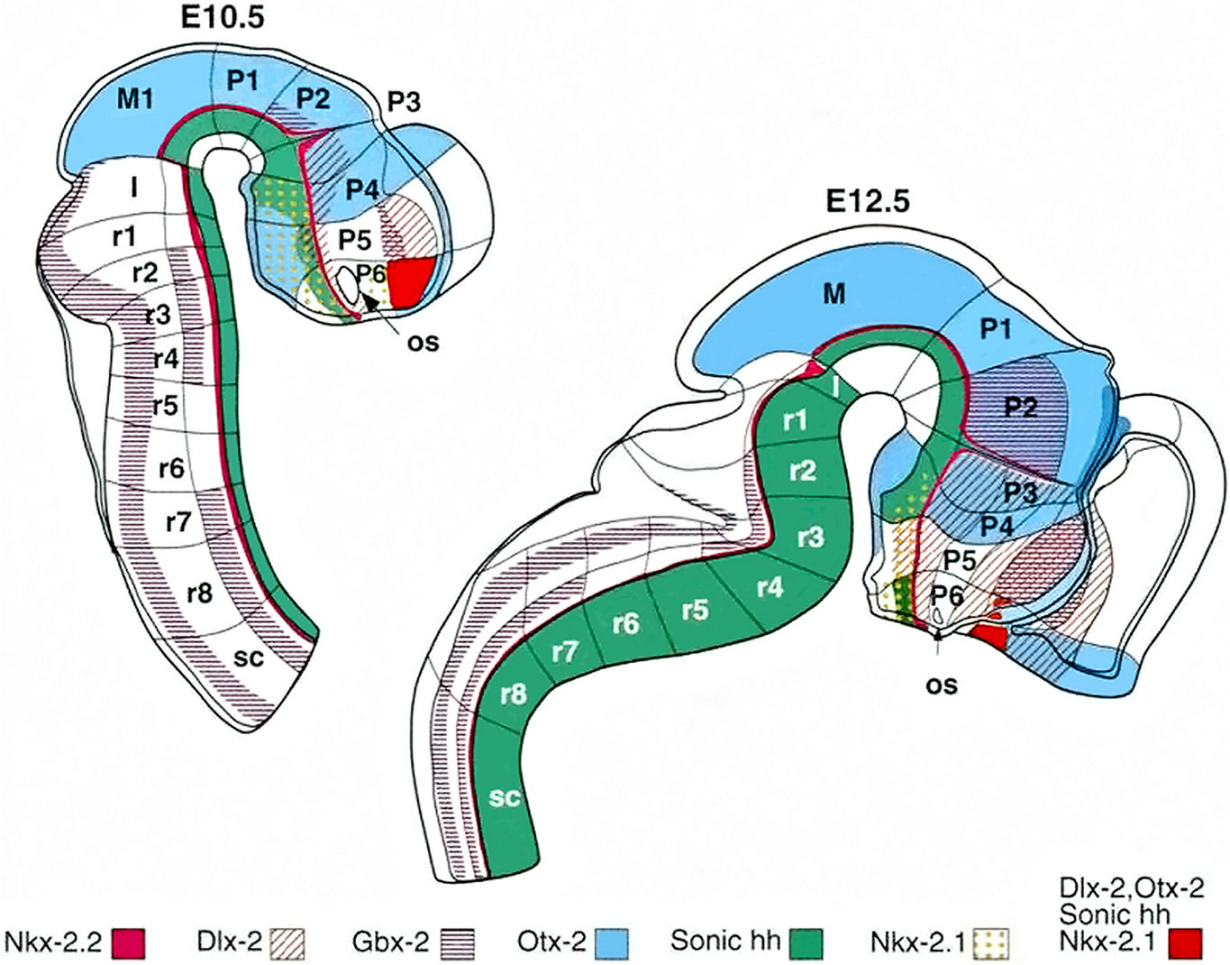
The prosomeric model of forebrain subdivision is based on gene expression patterns. The embryonic mouse brain can be divided in rhombencephalon–hindbrain (rhombomeres r1–r8), isthmus, mesencephalon, diencephalon (prosomeres p1–p3, from caudal to rostral), and the secondary prosencephalon (prosomeres p4–p6). The analyzed genes (*Dlx2*, *Gbx2*, *Nkx2-1*, *Nkx2-2*, *Otx-2*, and *Shh*) are expressed in restricted parts of the neuroepithelium. The hypothalamus is a part of two prosomeres, p4 and p5. According to the updated prosomeric model (Puelles and Rubenstein, 2015), these prosomeres are now two hypothalamo-telencephalic prosomeres (hp1 and hp2). The hp1 prosomere (former p4 prosomere) is called the peduncular hypothalamus (PHy), and the hp2 prosomere (former p5 prosomere) is the terminal hypothalamus (THy), which occupies the rostralmost part of the forebrain. H, rhombencephalon-hindbrain; I, isthmus; M, mesencephalon-midbrain; os, optic stalk; p, prosomere; r, rhombomere; sc, spinal cord; SP. Figure from the article by Rubenstein et al. (1994). Reprinted with permission from the American Association for the Advancement of Science.

These problems were resolved with the advent of molecular neurobiology and the discovery of genes involved in brain development and patterning. The experimental data and expression patterns of possible regulatory genes proved difficult to interpret within the columnar model as the expression patterns did not follow previously postulated borders of forebrain subdivisions. Thus, in an attempt to unite morphologic and gene expression data, Puelles and Rubenstein developed the prosomeric model (Rubenstein et al. 1994; Puelles and Rubenstein 2003; Puelles et al. 2013). The prosomeric model follows postulates of the previous neuromeric model of Bergquist and Källěn (1954, 1955) and proposes that the forebrain can be divided in transverse domains called prosomeres (shortened from “prosencephalic neuromeres”). Each of them may be further subdivided in longitudinal domains (i.e., the roof, alar, basal, and floor plate), creating a grid-like structure in which one “square” represents one basic morphogenetic unit (Rubenstein et al., 1994; Puelles and Rubenstein, 2003; Puelles et al., 2013; Fig. 1). This model is based on embryological evidence about the phylogenetically conserved segmental organization of the CNS in all vertebrates. Additionally, fate-mapping and transgenic animals resolved issues raised by older neuromeric models, thus experimentally proving concepts of prosomeric model. For instance, boundaries between proneuromeres of Bergquist and Källěn (1954, 1955) are retained in the adult brain as molecular boundaries between prosomeres (Puelles, 2021), and the sulcus limitans of His (1893a,b) is basically the molecularly defined alar–basal boundary in the prosomeric model (Puelles et al., 2012). However, there are opposing views regarding the number of prosomeres. Figdor and Stern (1993) analyzed the segmental organization of chick embryo and came to the conclusion that the hypothalamus and the ventral thalamus (now called the “prethalamus”; “prosomere p3” in the prosomeric model) make up one segment (neuromere D1), suggesting they have a common developmental origin. Furthermore, several recent articles reported gene expression patterns in the developing diencephalon and hypothalamus, which were difficult to interpret within the prosomeric model. These studies suggest that there is a set of genes with expression domains crossing proposed prosomeric boundaries, especially the hypothalamo-prethalamic boundary (Shimogori et al., 2010; Bedont et al., 2015; Newman et al., 2018). These inconsistencies should be resolved in the future as our knowledge about early hypothalamic patterning increases.

One of the biggest changes in the prosomeric model is the interpretation of the diencephalic and hypothalamic development. The hypothalamus had classically been perceived as a ventral longitudinal part of the diencephalon, as the authors assumed that the longitudinal axis of the forebrain continues in the telencephalon (Herrick, 1910; Kuhlenbeck, 1954; Reinoso-Suarez, 1966; Richter, 1966; Swanson, 2012). In the prosomeric model, the hypothalamus is separated from the diencephalon and comprises the most rostral part of the neural tube, lying rostrally to the diencephalon and ventrally to the telencephalon (Fig. 1).

## The Origin of STN Neurons and the Concept of Subthalamus

The new model of hypothalamic development has direct implications for our understanding of the origin of STN neurons, so we will re-examine existing studies in the context of the prosomeric model.

**Figure 2.**
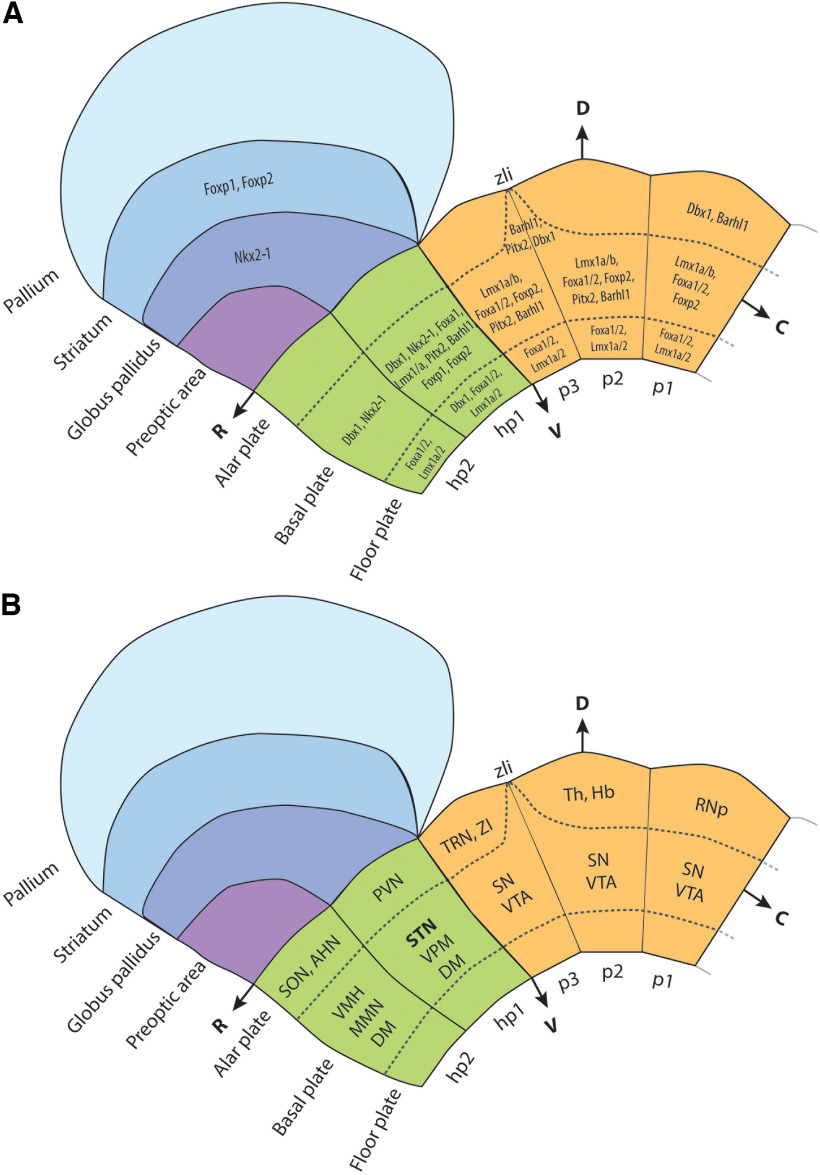
A schematic representation of forebrain subdivisions, expression patterns of TFs, and major hypothalamic and diencephalic nuclei originating from each prosomere. ***A***, Forebrain subdivisions and expression patterns of TFs according to the prosomeric model. The developing STN can be found in the basal plate of the hp1 prosomere. Notice how the STN shares a set of TFs with other hypothalamic and diencephalic nuclei, as well as parts of the subpallium (i.e., basal ganglia). The telencephalon and the hypothalamus comprise the secondary prosencephalon. The diencephalic and the hypothalamic prosomeres have alar, basal, and floor plates. Also, in the prosomeric model, the hypothalamus is the rostralmost prosencephalic domain, and the STN is placed in the retromammillary area of the hp1 prosomere, the ventralmost part of the hypothalamus. ZLI is a transverse border between p2 and p3 prosomere. ***B***, major hypothalamic and diencephalic nuclei originating from each prosomere. Parts of the substantia nigra and ventral tegmental area have diencephalic origin (Puelles, 2019), so they are parts of diencephalic prosomeres p1–p3. The dorsomedial nucleus originates from both peduncular and terminal hypothalamic domains. AHN, Anterior hypothalamic nucleus; DM, dorsomedial nucleus; Hb, habenula; MMN, mammillary nuclei; POA, preoptic area; PVN, paraventricular nucleus; RNp, parvocellular part of nucleus ruber; SN, substantia nigra; SON, supraoptic nucleus; Th, thalamus; TRN, thalamic reticular nucleus; VMH, ventromedial nucleus; VPM, ventral premammillary nucleus; C, Caudal; D, dorsal; R, rostral; V, ventral.

Part of the problem with studying the developmental origin of STN lies in the terminological confusion as terms “hypothalamus” and “subthalamus” have historically been used interchangeably or their anatomic borders were not clearly defined (Puelles et al., 2012). When comparing classical studies with the prosomeric model, the term “subthalamus” refers to the peduncular hypothalamus [hypothalamic prosomere 1 (hp1)], and the term “hypothalamus” refers just to the terminal hypothalamus (hp2; Fig. 2*A*; Reinoso-Suarez, 1966; Richter, 1966). Upon closer inspection of the literature, another problem we encountered is the lack of clear distinction between the subthalamus and the “subthalamic nucleus.” Furthermore, classic neuroanatomists had their own interpretations of what the subthalamus encompasses. The ZI and the GP have both been included in the subthalamus, whereas modern fate-mapping and molecular studies ascribed different developmental origins for these nuclei, the prethalamus for the former, and the medial ganglionic eminence (MGE) for the latter (Reinoso-Suarez, 1966; Richter, 1966; Nóbrega-Pereira et al., 2010; Puelles et al., 2012). To avoid this confusion, Puelles et al. (2012), Puelles and Rubenstein (2015), and Puelles (2019) advocate that the term “subthalamus” should be abandoned as it has no developmental basis.

The STN was first described to arise from the mammillary region of the hypothalamus (Gilbert, 1935). In the human brain, the first appearance of the “subthalamic area” within the hypothalamus is described at 33–35 d of gestation, but the “subthalamic nucleus” is not unambiguously defined (Müller and O’Rahilly, 1988). At 44–51 d of gestation, the STN can be seen near the mesencephalon and the mammillary body, with cellular strands connecting the nucleus with the supramammillary recess, its presumed place of origin (Müller and O’Rahilly, 1990).

The question of the origin of STN neurons was explored in the Chinese hamster using [^3^H]-thymidine autoradiography. The study by Keyser (1973) described for the first time the tangential rostrodorsal migration of STN neurons from the supramammillary recess toward the tel-diencephalic border. The Keyser’s term “regio subthalamica” can be discerned from embryonic day 12 (E12) and refers to the area behind the mammillary recess, whereas the STN is recognizable from E15, with the majority of neurons born from E13 to E18. Similar experiments in rats demonstrated that STN originates from the germinative zone near the mammillary recess from which neurons migrate radially, then tangentially and dorsally along the marginal layer of the ventral thalamus (Marchand, 1987). When analyzing the results of these studies, it becomes apparent that the change in forebrain axis proposed by the prosomeric model added to terminological confusion. The “(supra)mammillary recess” that Müller and O’Rahilly (1990), Keyser (1973), and Marchand (1987) describe is actually the retromammillary area in the prosomeric model. The retromammillary area is a part of a broad mammillary region that can be divided in two rostrocaudal parts, with the mammillary area being the rostral part from which the mammillary nuclei arise, and the retromammillary area being the caudal part. In the ventrodorsal (V-D) direction, the retromammillary area comprises the floor and basal plate of the hypothalamic prosomere 1 (hp1; Fig. 2*A*).

The STN neurons in rats are generated between E12 and E15 with most neurons migrating between E14 and E15 in an “outside–in” fashion, meaning that the early-born neurons settle in the laterodorsal part of the STN, and the late-born neurons settle in the ventromedial part (Altman and Bayer, 1979a,b). Interestingly, these authors placed the STN in the ventral thalamus (which corresponds to the prosomere p3, now called the prethalamus) instead of the hypothalamus, claiming the nucleus originates from the ventrocaudal diencephalic neuroepithelium and later migrates laterodorsally over the fibers of the cerebral peduncle (Altman and Bayer, 1979b).

Based on everything discussed in previous paragraphs, the main question is whether the STN has hypothalamic or diencephalic origin. The confusion about the place of origin of the STN arose from the differences in the interpretation of the hypothalamus as a developmental unit. The columnar model proposes that the hypothalamus is a ventralmost diencephalic domain; whereas the prosomeric model states that the hypothalamus is a separate developmental unit (Puelles and Rubenstein, 2015). The STN was traditionally attributed to the diencephalon because of its anatomic position in the adult brain and functional connectivity with the basal ganglia circuitry. However, the dorsalward tangential migration of STN from the retromammillary area of the hypothalamus was noted in early descriptive works and was corroborated using modern tracing experiments (Gilbert, 1935; Keyser, 1973; Marchand, 1987; Müller and O’Rahilly, 1990; Martin et al., 2004; Skidmore et al., 2008). Contrary to that, authors like Altman and Bayer (1979b) placed the origin of STN in the ventral thalamus (prethalamus), but the hypothalamic and prethalamic lineages can now easily be distinguished by analyzing gene expression patterns. As we will discuss in the following paragraphs, the STN clearly expresses several basal/hypothalamic markers, which provides strong evidence against a diencephalic–prethalamic origin of the STN and supports retromammillary/hypothalamic origin (Vue et al., 2007; Kee et al., 2017; Guo and Li, 2019; Kim et al., 2020; Mickelsen et al., 2020; Wallén-Mackenzie et al., 2020).

## Induction and Early Patterning of Hypothalamic Primordium

The induction of the neural plate and the patterning of the forebrain are complex processes, so the in-depth discussion of these topics is beyond the scope of this review. In the following paragraphs, we will summarize key findings regarding the early forebrain development. The hypothalamus, along with the rest of the forebrain, arises from a part of the ectoderm that thickens under the influence of the underlying mesoderm and forms the neural plate (Bronner-Fraser and Fraser, 1997). Ectodermal cells destined to become the forebrain have to go through neural induction, specification of an anterior character, and initial regionalization in anteroposterior (A-P) axis (for review, see Stern, 2001, 2006; Wilson and Houart, 2004). However, the exact sequence of events during the early forebrain development (from gastrulation to late neural plate) is still a controversial subject (Stern, 2002). As Stern (2001) argues, perhaps more complex mechanisms (e.g., prepatterning of the neural plate or timing of exposure to inductive signals) govern these processes that remain to be experimentally proven.

The first specification of mammalian neural tissue occurs during gastrulation and is initiated through interactions between a primary organizer (for the definition of an organizer, see Anderson and Stern, 2016) called the node and the surrounding ectoderm (Rubenstein et al., 1998; Wilson and Houart, 2004). Accumulated evidence point to neural induction beginning before the formation of the node, so the neural fate is promoted by fibroblast growth factor (FGF) signaling and later sustained by bone morphogenetic protein (BMP) antagonism (Wilson and Houart, 2004; Vieira et al., 2010). It is crucial for proper forebrain development that the anterior neural tissue retains its identity and repels caudalizing influences. In chick and mouse embryo, the node and its derivative, the prechordal mesendoderm, protect the anterior neural plate against caudalizing influences of signaling molecules like Wingless type proteins (WNTs), FGFs, BMPs, and retinoic acid (Stern, 2001; Wilson and Houart, 2004; Cajal et al., 2014). At this early stage of forebrain development, these secreted factors create a basic A-P pattern, which will later be refined by local signals from the neural tissue. This A-P pattern creates transverse segments with differential competence to respond to the same inductive signal (Shimamura and Rubenstein, 1997; Rubenstein et al., 1998). Moreover, the neural plate (and neural tube) is patterned in mediolateral (M-L; or V-D in the neural tube) direction under the influence of non-neural tissue (Ruiz i Altaba, 1994; Rubenstein et al., 1998; Vieira et al., 2010). The grid-like image of neural plate that develops after A-P and M-L (V-D) patterning served as a basis for the prosomeric model of brain development (discussed in previous paragraphs). In the prospective forebrain, the axial mesendoderm (the prechordal plate) specifies medial/ventral cell fate, whereas lateral/dorsal cell fate is imparted by the influence of non-neural ectoderm (Shimamura and Rubenstein, 1997; Rubenstein et al., 1998). The non-neural ectoderm specifies neural crest cells through planar interactions with the neighboring neural plate (Barembaum and Bronner-Fraser, 2005; Basch and Bronner-Fraser, 2006). Furthermore, non-neural ectodermal cells precede the development of the anterior neural ridge, a local organizer (“secondary” organizer) that has a role in the regionalization of the telencephalon (Shimamura and Rubenstein, 1997; Cajal et al., 2014).

Secondary organizers form as specialized parts of the neuroepithelium that act as molecular boundaries, thus preventing intermixing of cells of different lineages (Vieira et al., 2010; Kiecker and Lumsden, 2012). By secreting morphogenes, secondary organizers further influence brain patterning, resulting in the development of brain vesicles (Rubenstein et al., 1998; Vieira et al., 2010). Morphogens are locally secreted molecules with the ability of organizing surrounding cells into patterns (Gurdon and Bourillot, 2001; Rogers and Schier, 2011). This is accomplished through their concentration gradient, so cells change their fate in response to the sensed level of morphogen (e.g., cells exposed to high morphogen concentrations activate different transcriptional programs than those exposed to low concentrations; Schmidt et al., 2008; Rogers and Schier, 2011). Concentration gradient formation can be explained by a synthesis, diffusion, and degradation model (Rogers and Schier, 2011). The idea of the concentration gradient for a morphogen is usually associated with positional information (Wolpert, 1989; Cooke, 1995; Lawrence and Struhl, 1996; Gurdon and Bourillot, 2001). However, the concentration gradient might not be solely responsible for the effects of morphogen activity: the time of exposure to a morphogen and cellular context, the so-called “sequential cell context model,” might also play a role (Pagès and Kerridge, 2000). Another interesting theory is that morphogens act on groups of cells that have gained collective properties through homotypic interactions and have acquired a level of competence to respond to secreted signals (Chandebois and Faber, 1983; Gass and Hall, 2007). According to this theory, a group of cells is able to differentiate through periodic autonomous developmental progressions that are intermittently influenced by signals from other parts of the tissue, as long as the cells are not dissociated. Also, this “collective behavior” could function as to ensure that the information (e.g., morphogenetic signal) is delivered to cells that are far from the source signal (Chandebois and Faber, 1983; Gass and Hall, 2007).

In relation to the forebrain patterning, the prevailing theory is that the concentration gradients of secreted morphogens [Sonic hedgehog (SHH), WNTs, BMPs] create a positional map of the neural tube, thus creating subdivisions in the dorsoventral and anteroposterior axes (Rubenstein et al., 1998; Vieira et al., 2010; Burbridge et al., 2016; Xie and Dorsky, 2017). These subdivisions are later compartmentalized into smaller progenitor domains during the process of regionalization. The progenitor domains are specified by cross-repressive interactions of transcription factors in the neuroepithelium. A part of the neuroepithelium with a shared set of TFs later generates neuronal subpopulations with common traits like neurotransmitter phenotype or axonal connections (Alvarez-Bolado, 2019; Diaz and Puelles, 2020). In the following paragraphs, we will discuss morphogens (SHH, FGFs, WNTs/WNT antagonism) secreted by primary and secondary organizers, and comment on their role in the patterning of the hypothalamic primordium. In addition, the reader can find the detailed description of hypothalamic induction and patterning in reviews by Bedont et al. (2015), Burbridge et al. (2016), Xie and Dorsky (2017), Alvarez-Bolado (2019), Diaz and Puelles (2020). However, these studies should be interpreted with a caveat. The authors used different models of hypothalamic development (i.e., prosomeric or modified columnar model), so the reader should be aware of the difference in the brain axis when interpreting the direction of morphogen activity.

### Sonic hedgehog signaling

*Shh* is a morphogen secreted from the mesendodermal tissue—the notochord and the prechordal plate, which confers medial (ventral) identity to the neural plate and consequently the neural tube (Shimamura and Rubenstein, 1997; Rubenstein et al., 1998; Wilson and Houart, 2004). Experimental evidence points to a crucial role of *Shh* in medial (ventral) patterning of the entire mouse CNS (Chiang et al., 1996). Shh is expressed throughout the axial mesendoderm (notochord and prechordal plate), yet it can induce different transcriptional programs at different A-P positions along the neural tube. For example, *Shh* from the notochord induces the expression of *Nkx6-1* posteriorly, whereas prechordal *Shh* induces *Nkx2-1* expression in the anterior neural tube (Shimamura and Rubenstein, 1997; Rubenstein et al., 1998; Vieira et al., 2010).

SHH plays an indispensable role in both the early induction and patterning of the hypothalamic primordium, and its later differentiation and growth (Blaess et al., 2014; Zhang and Alvarez-Bolado, 2016). However, there is still dispute over the primary source of *Shh* and the induction of the floor plate. Based on experiments in chick embryo, two theories have been proposed. One theory proposes that the floor plate differentiates under *Shh* signaling from the notochord (Dodd et al., 1998; Placzek et al., 2000), while the other theory proposes that the floor plate and the notochord develop from common precursors originating from the organizer. The floor plate cells are later simply added in the ventral midline as a consequence of the elongation of the embryo in the A-P axis (Le Douarin and Halpern, 2000; Kiecker and Lumsden, 2012). Another issue is the question of induction of the hypothalamic floor plate. Again, there are two possible explanations, which are discussed at length in the studies by Puelles and Rubenstein (2015), Fu et al. (2017), Fu et al. (2019), and Diaz and Puelles (2020). Briefly, one explanation is that the hypothalamic floor plate differentiates under SHH influence from the underlying prechordal mesoderm (Burbridge et al., 2016; Fu et al., 2017, 2019), while the other theory proposes that SHH secreted from the notochord induces the hypothalamic floor plate. Hypothalamic floor plate then acts as a secondary organizer and expresses *Shh,* thus influencing the patterning of the basal plate (Diaz and Puelles, 2020).

In summary, the basal plate is probably specified by cooperative *Shh* signaling from non-neural (prechordal mesoderm and notochord) and neural (floor plate) sources (García-Calero et al., 2008; Fu et al., 2019; Diaz and Puelles, 2020). On the other hand, the hypothalamic primordium acts as a neuroepithelial source of *Shh* and the majority of hypothalamic cells belongs to the *Shh* lineage (Xu et al., 2008; Szabó et al., 2009; Shimogori et al., 2010; Alvarez-Bolado et al., 2012; Blaess et al., 2014; Zhang and Alvarez-Bolado, 2016). In addition to these hypothalamic progenitors, the *Shh^+^* progenitors can also be found in the mesencephalic floor plate. These cells will constitute the dopaminergic neurons of the adult ventral tegmental area (VTA) and SN (Joksimovic et al., 2009; Blaess et al., 2011).

Another source of *Shh* signaling in the developing forebrain is the zona limitans intrathalamica (ZLI), also called the mid-diencephalic organizer (Kiecker and Lumsden, 2004). This secondary organizer is located at the transverse border between prosomere 2 (p2; thalamus) and p3 (prethalamus), thus influencing the A-P patterning and nucleogenesis in the diencephalon (Scholpp and Lumsden, 2010; Martinez-Ferre and Martinez, 2012). The exact mechanism by which ZLI forms is still being debated and different mechanisms have been proposed for amniotes and anamniotes (for review, see Vieira et al., 2005; Vieira and Martinez, 2006; Scholpp and Lumsden, 2010). Both hypothalamic and diencephalic (i.e., thalamic) primordia are under *Shh* influence. However, there is a significant difference between them, indicating their separate developmental origin. The hypothalamus is under the influence of both non-neural *Shh* signals (from the notochord and the prechordal mesoderm) and neural *Shh* (from the floor and basal plate), whereas the thalamus is only under the influence of non-neural *Shh* morphogenetic signals from the ZLI, thus having no cells of *Shh* lineage (Zhang and Alvarez-Bolado, 2016).

*Shh* expression can also be found in other parts of the brain. The expression patterns of both mRNA and protein products of *Shh* signaling cascade have been studied in rodent models and human developing brain. In adult rat brain, *Shh* mRNA has been found in the GP, whereas STN and several hypothalamic nuclei express the SHH receptor *Ptch* (Traiffort et al., 1999). Similar expression patterns have been reported in the developing human brain (Memi et al., 2018). In addition to influencing hypothalamic neuronal lineages, *Shh* exerts influence on the development of neurons in MGE and lateral ganglionic eminence (LGE) destined for striatum, pallidum, and cortex (Kohtz et al., 1998).

To summarize, early non-neural expression of SHH is important for the patterning of the entire forebrain. However, hypothalamic and telencephalic neuronal expression of SHH, coupled with its absence from the thalamus, supports the notion that hypothalamic primordium is a distinct developmental site and not a part of diencephalon.

### Wnt signaling

Wnts are important morphogens that posteriorize the neural plate, thus influencing the initial A-P patterning (Wilson and Houart, 2004; Vieira et al., 2010). Wnts and Wnt antagonists create a gradient of Wnt activity, which is translated into positional information along the A-P axis of the neural tube (Wilson and Houart, 2004; Xie and Dorsky, 2017). In later stages of forebrain development, Wnts act as important regulators of anteroposterior patterning (Bedont et al., 2015; Burbridge et al., 2016). Moreover, Wnts influence somitogenesis, and induction of neural crest cells, and their delamination and migration (Schmidt et al., 2008). Wnt signaling specifies hypothalamic and diencephalic fates, characterized by *Foxd1* expression, while Wnt antagonism specifies *Foxg1*^+^ anterior telencephalic fates (Blackshaw et al., 2010; Burbridge et al., 2016; Newman et al., 2018). In zebrafish embryos, Wnt signaling through *Wnt8b* plays a role in neurogenesis in the posterior hypothalamus (Lee et al., 2006). Similar mechanism was described in mouse embryo where gain-of-function and loss-of-function studies of Wnt/β-catenin signaling pathway revealed its role in posteriorizing the hypothalamic primordium. Specifically, loss-of-function mutants exhibited a reduced size of the posterior hypothalamus (evidenced by loss of supramammillary and mammillary markers; e.g., *Foxa1*, *Irx5*, *Foxb1*, *Sim1*), while gain-of-function mutants had an expanded expression domain of posterior and premammillary markers (*Pitx2*, *Lhx5*), with reduced expression domains of a subset of anterior markers (Newman et al., 2018). Furthermore, *Wnt8b* has an evolutionary conserved expression in caudal, mammillary hypothalamus (or ventralmost hypothalamic region in the prosomeric model; Xie and Dorsky, 2017). This mammillary and retromammillary expression domain was also described in human embryos during early gestation (Lako et al., 1998). Diaz and Puelles (2020) hypothesize that the *Wnt8b* expression could mark the cells of the hypothalamic ventricular organ, a possible (yet still poorly understood) secondary organizer involved in patterning of the basal hypothalamus, namely its tuberal and mammillary part. The experiments in chick embryos provided evidence that *Wnt8b* is also an important marker of ZLI, thus indicating its role in diencephalic patterning and corroborating the prosomeric organization of the forebrain (Garda et al., 2002; Garcia-Lopez et al., 2004).

In summary, *Wnt*s are important factors in patterning of both hypothalamus and diencephalon, with *Wnt8b* serving as a marker of a possible secondary organizer involved in the development of the hypothalamus.

### Fibroblast growth factor signaling

FGF family member *Fgf8* initiates neural induction and later acts as an inductive signal capable of eliciting different molecular responses at different levels of the A-P axis of the neural tube (e.g., anterior expression of *Foxg1*/*BF-1*, posterior expression of *En2*; Rubenstein et al., 1998; Wilson and Houart, 2004; Vieira et al., 2010). For instance, *Fgf8* is implicated in the development of the isthmic organizer located at the mid-hindbrain boundary, a secondary organizer important for the generation of dopaminergic and serotonergic neurons (Hynes and Rosenthal, 1999). Moreover, *Fgf8* can be found in the anterior neural ridge, a secondary organizer that influences telencephalic patterning, which later transforms into the hypothalamo-telencephalic roof plate (Rubenstein et al., 1998; Diaz and Puelles, 2020). The anterior neural ridge had classically been perceived as having a rostralizing effect on the prosencephalon, but, within the prosomeric model, this organizer has a dorsalizing effect on both the hypothalamic regions and the telencephalon (Diaz and Puelles, 2020). In the prosomeric model, the rostralmost part of the hypothalamo-telencephalic complex is acknowledged as another organizer domain with *Fgf8* secretion, called the acroterminal domain. The *Fgf8* signal from this organizer spans from the end of the mammillary floor plate to the place of the future anterior commissure (Ferran et al., 2015; Diaz and Puelles, 2020; López-González et al., 2021). Together with *Shh*, *Fgf8* plays a role in the morphogenesis of midline structures, and the splitting of the eye primordium and the telencephalic vesicle (McCabe et al., 2011; Dubourg et al., 2016).

Experiments with *Fgf8* mutant mice elucidated the role of *Fgf8* in prosencephalic/telencephalic patterning. It seems that *Fgf8* promotes rostroventral telencephalic fates, since *Fgf8* hypomorphic mice have a caudalized anterior telencephalon (as evidenced by the expanded expression zones of *Emx2*, *Otx2*, *COUP-TF1*). Moreover, the ventral telencephalon (i.e., the septum and the ganglionic eminences) is lost (Garel et al., 2003; Storm et al., 2006), and hypothalamo-pituitary malformations are also present (Brooks et al., 2010; McCabe et al., 2011; Diaz and Puelles, 2020). *Fgf8* secreted from the acroterminal domain might influence the development of the retromammillary area and its derivatives, mainly the STN and the ventral premammillary nucleus, primarily by exerting trophic influence on migrating neuronal populations of these nuclei (López-González et al., 2021). This is substantiated by observations that mouse *Fgf8* hypomorphs exhibit no migration deficit, whereas cellular populations of ventral premamillary nucleus and STN are severely decreased (López-González et al., 2021).

In conclusion, *Fgf8* secreted from the anterior neural ridge and the acroterminal domain has dorsalizing and rostralizing effects (respectively) on the hypothalamic primordium.

## Transcription Factors in the Developing and Adult Subthalamic Nucleus

Unique combinations of morphogens induce the expression of transcription factors, which regulate the further development and specification of the STN area. The concept of genomic regulatory networks (Beccari et al., 2013) helps us to understand how the molecular complexity of the forebrain emerges. The TFs higher up in the hierarchy control the specification of the progenitors, and hierarchically lower TFs are expressed in postmitotic cells of restricted lineage (Beccari et al., 2013; Alvarez-Bolado, 2019). These postmitotic TFs also regulate the migration, axon guidance, and acquisition of other phenotypic characteristics (Alvarez-Bolado, 2019). The TFs presented here have been selected based on the data available from the literature, observed expressions in the *Allen Brain Atlas* of mouse and human brain development, as well as data on differentially expressed genes from human prenatal microarray studies. We organized TFs according to the position within the genomic regulatory network (i.e., neural progenitors, postmitotic neurons). All data presented here pertain to the developing mouse brain, if not specified otherwise.

### Transcription factors expressed in neural progenitors

#### Developing brain homeobox protein 1

**Figure 3.**
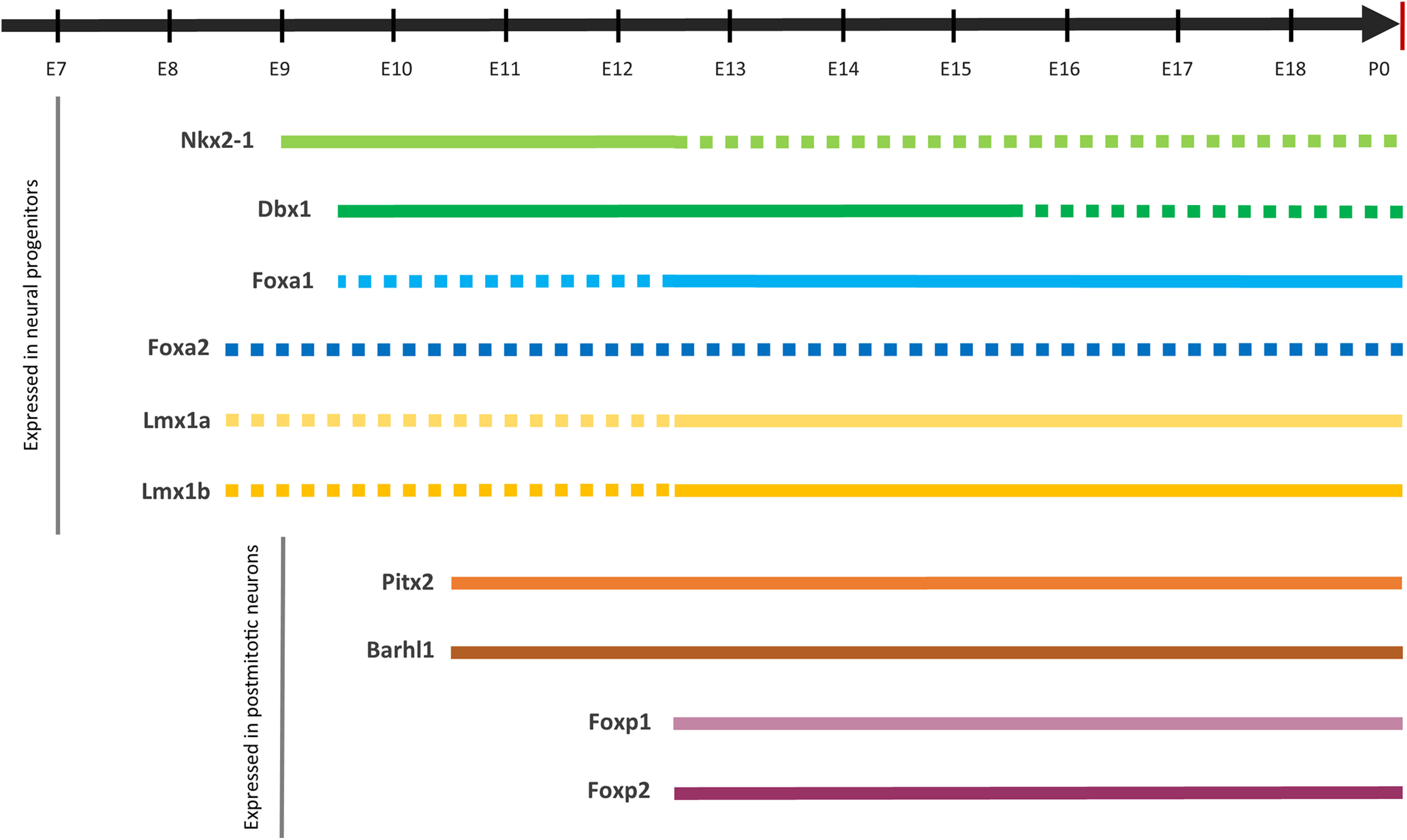
Gene expression timelines during mouse embryonic development. Full lines represent mRNA expression detected by ISH in the hypothalamic retromammillary area/developing STN. Dashed lines represent the expression detected in other parts of the developing forebrain. For *Foxa2*, dashed lines indicate there are no conclusive results about its expression pattern in the developing STN (see text).

Developing brain homeobox protein 1 (*Dbx1*) belongs to the homeobox family of transcription factors and can be detected in the cephalic primordium around E8.5 (Causeret et al., 2011). In the hypothalamic and diencephalic primordium, *Dbx*1 expression was detected at approximately E9.5 (Lu et al., 1992; Shoji et al., 1996). From E10.5 to E15.5, its signal can be detected in the ventricular zone of the basal hypothalamus, mammillary and retromammillary area, ZLI, and the pretectum (p1; Figs. 2*A*, 3, Table 1; Shoji et al., 1996). As progenitors differentiate and migrate away from the ventricular zone, *Dbx1* is downregulated (Sokolowski et al., 2016). During the embryonic period, *Dbx1* knockouts exhibited a loss of *Npy* expression in the arcuate nucleus, along with the loss of *Pmch* and *Hcrt* expression in the lateral hypothalamus. Therefore, *Dbx1* is an important determinant of orexigenic neurons in the arcuate nucleus and lateral hypothalamus, constitutive parts of feeding and stress response circuits (Sokolowski et al., 2015; Alvarez-Bolado, 2019).

**Table 1:** A Summary of the discussed transcription factors, their mRNA expression zones in the rodent and human brain, and the list of papers in which this information can be found

Transcription factor	Species	Expression zone in the brain	Reference
Dbx1	Embryonic mouse Adult mouse	Mesodiencephalic floor plate Mammillary and retromammillary area, ZLI, pretectum, ARC, LH	Nouri and Awatramani, 2017; Kee et al., 2017 Lu et al., 1992; Shoji et al., 1996; Sokolowski et al., 2015, 2016
Nkx2-1	Embryonic mouse and rat Fetal human Adult mouse and rat	MGE, preoptic area, basal hypothalamic neuroepithelium MGE, preoptic area Cortical and striatal interneurons, prototypic neurons in the GPe, ARC, VMH, and MMN	Kimura et al., 1996; Sussel et al., 1999; Nakamura et al., 2001; Flames et al., 2007; Xu et al., 2008; Puelles and Rubenstein, 2015; Murcia-Ramón et al., 2020 Pauly et al., 2013 Nakamura et al., 2001; Magno et al., 2009; Flandin et al., 2010; Abdi et al., 2015; Dodson et al., 2015
Foxa1	Embryonic mouse Adult mouse	Mesodiencephalic floor plate, retromammillary area STN, SMN, VPM, VTA, and PH	Lin et al., 2009; Mavromatakis et al., 2011; Díaz et al., 2014; López-González et al., 2021 Gasser et al., 2016
Lmx1a	Embryonic mouse Embryonic and adult mouse	Mesodiencephalic floor plate, roof plate and derivatives SMN, VPM, STN, VTA, SNc	Manzanares et al., 2000; Lin et al., 2009; Doucet-Beaupré et al., 2015 Failli et al., 2002; Zou et al., 2009; Hoekstra et al., 2013
Lmx1b	Embryonic mouse Embryonic and adult mouse	Mesodiencephalic floor plate STN, PSTN, VPM, PH, LH, SNc, raphe nuclei	Lin et al., 2009; Doucet-Beaupré et al., 2015 Asbreuk et al., 2002; Dai et al., 2008; Skidmore et al., 2008
Pitx2	Embryonic mouse Adult mouse	Ventral mesencephalon, basal plate p1–p3, basal plate of hp1, ZLI, ZI; alar plate of the mesencephalon STN, ZI, SMN, MMN, pituitary gland, superior colliculus	Muccielli et al., 1996; Martin et al., 2002, 2004; Kim et al., 2020 Martin et al., 2002, 2004; Skidmore et al., 2008; Waite and Martin, 2015
Barhl1	Embryonic mouse Fetal human	Basal plate p1–p3, retromammillary and mammillary area, ZLI, pretectum, inferior and superior colliculi, and rhombic lips Basal plate p1–p3, retromammillary and mammillary area, ZLI, pretectum, inferior and superior colliculi, rhombic lips	Bulfone et al., 2000; Rachidi and Lopes, 2006 Lopes et al., 2006
Foxp1	Embryonic mouse and rat Adult mouse and rat Fetal human	Cortical plate, LGE, striatal projections neurons, hypothalamic basal plate, CA1 field of the hippocampus, subiculum, cerebellum Cortical layers 3–5, striatum, CA1 field of the hippocampus, subiculum, SMN, MMN, STN, cerebellum Striatum, thalamus, STN, SN	Ferland et al., 2003; Takahashi et al., 2003; Tamura et al., 2004; Co et al., 2020; Kim et al., 2020 Lai et al., 2003; Takahashi et al., 2003; Tamura et al., 2004; Co et al., 2020 Teramitsu et al., 2004; Co et al., 2020
Foxp2	Embryonic mouse and rat Adult mouse and rat Fetal human	Cortical plate, LGE, striatal projection neurons, hypothalamic basal plate, thalamus, amygdala, and cerebellum Cortical layer 6, striatum, thalamus, amygdala, SMN, MMN, SN, STN, cerebellum, and GP Striatum, thalamus, STN, SN, and GPe	Ferland et al., 2003; Lai et al., 2003; Takahashi et al., 2003; Co et al., 2020 Ferland et al., 2003; Takahashi et al., 2003; Fong et al., 2018; Co et al., 2020 Lai et al., 2003; Teramitsu et al., 2004; Co et al., 2020

ARC, Arcuate nucleus; LH, lateral hypothalamus; PH, posterior hypothalamus; PSTN, parasubthalamic nucleus; SMN, supramammillary nucleus; MMN, mammillary nuclei; VPM, ventral premammillary nucleus. Other abbreviations are the same as the ones used in Figure 2.

*Dbx1* is also identified as one of the TFs regulating floor plate progenitors. Initial studies analyzing mesencephalic dopaminergic progenitors in the floor plate could not establish a clear-cut rostral border of the domain, thus prompting some authors to term the entire area mesodiencephalic continuum (Puelles and Rubenstein, 2015; Nouri and Awatramani, 2017; Puelles, 2019). This raised a question of whether glutamatergic STN and dopaminergic neurons share a common origin, as the rostral border could extend into hypothalamic territory. Recent experiments in mouse embryos proposed that the floor plate could be divided into at least two subdomains in A-P axis. One of these domains is anterior, expresses *Dbx1*, and gives rise to a glutamatergic population (STN included), while the posterior domain, characterized by *En1*, gives rise to dopaminergic neurons of SN pars compacta (SNc) and VTA (Nouri and Awatramani, 2017). This finding was further supported by the results of single-cell profiling that have identified *Dbx1* in *Lmx1a^+^* glutamatergic cells of the mesodiencephalic continuum (Kee et al., 2017). Based on these results, we can conclude that glutamatergic and dopaminergic neurons, although generated in bordering domains, could be distinguished by their different transcriptional profiles.

#### Nk2 homeobox 1

Nk2 homeobox 1 [*Nkx2-1* (also known as *TTF-1*)] is another TF belonging to the homeobox gene family. In the developing mouse brain, *Nkx2-1* expression was first detected at approximately E8.75 (Shimamura et al., 1995, 1997). After E9, the expression can be detected in two domains, one occupying the subpallium—the MGE and preoptic area—and the other occupying the hypothalamic basal plate (Figs. 2*A*, 3, Table 1; Sussel et al., 1999; Marín et al., 2000; Flames et al., 2007; Xu et al., 2008).

The hypothalamic basal plate has been defined as a *Shh^+^*/*Nkx2-1^+^* territory (Shimamura et al., 1997; Puelles et al., 2012). *Nkx2-1^+^* progenitors can be found in the terminal and peduncular hypothalamus, while *Nkx2-1^+^* neurons contribute to adult arcuate nucleus, ventromedial and dorsomedial nuclei, and the mammillary nucleus, as demonstrated in both mice and rats (Nakamura et al., 2001: Puelles et al., 2012; Puelles and Rubenstein, 2015; Alvarez-Bolado, 2019; Murcia-Ramón et al., 2020; Barbier and Risold, 2021). The *Nkx2-1*-null mice have an abnormal hypothalamic anatomy, with many hypothalamic nuclei missing or underdeveloped (Kimura et al., 1996). Single-cell profiling of these mutants corroborated previous findings and established *Nkx2-1* as a repressor of prethalamic identity (Kim et al., 2020). Curiously, it appears that *Nkx2-1* is absent from the retromammillary area (Puelles et al., 2012; Puelles and Rubenstein, 2015). Recent experiments with E18.5 *Nkx2-1^cre/+^;tdTomato^flox/+^* mouse embryos actually established that the STN remains positive for red fluorescent protein, while NKX2-1 protein was absent, suggesting that the cells migrating away from the retromammillary area silence the *Nkx2-1* expression, similarly as tangentially migrating cortical interneurons (Sussel et al., 1999; Marín et al., 2000; Murcia-Ramón et al., 2020). The downregulation most likely occurs at approximately E12.5 as at that time point no *Nkx2-1* can be observed in the ventrocaudal hypothalamus (Murcia-Ramón et al., 2020).

The subpallial expression zone was detected in both mouse and human embryos. In human embryos, the *Nkx2-1* subpallial expression zone was detected in the late embryonic period by *Dlx2/Nkx2-1* colabeled cells in the MGE and preoptic area (Pauly et al., 2013). The MGE is the origin of striatal and cortical GABAergic and cholinergic interneurons. Striatal interneurons retain Nkx2-1 expression, while cortical interneurons downregulate *Nkx2-1* expression as they start their tangential migration toward the cerebral cortex (Nóbrega-Pereira et al., 2008). These interneurons can be identified and traced by the expression of *Lhx6* (downstream target of *Nkx2-1*) and various markers (e.g., parvalbumin, somatostatin, calbindin; Sussel et al., 1999; Marín et al., 2000; Flames et al., 2007; Xu et al., 2008; Magno et al., 2009; Flandin et al., 2010; Malt et al., 2016). *Nkx2-1* expression was retained in adult globus pallidus, a GABAergic nucleus with neuronal populations originating from the ganglionic eminences and preoptic area (Fig. 2*B*; Xu et al., 2008; Magno et al., 2009; Flandin et al., 2010; Nóbrega-Pereira et al., 2010). In mice, *Nkx2-1* has now been acknowledged as a marker of pallidal neurons originating from the MGE, expressing parvalbumin, projecting to the STN, and having a high-frequency firing rate (Abdi et al., 2015; Dodson et al., 2015). Interestingly, a recent study of the adult human STN described the expression of NKX2-1 in the STN, along with parvalbumin and calretinin (Bokulić et al., 2021). As mentioned before, postmitotic neurons destined to populate the mouse STN downregulate *Nkx2-1*, so the observed difference between human and mouse STN could indicate the existence of an additional neuronal subclass or a species-specific mechanism regulating its expression.

In summary, *Nkx2-1* is important for the specification of many neuronal classes. However, not all postmitotic neurons retain *Nkx2-1* expression (e.g., cortical interneurons). Evidence points to the conclusion that *Nkx2-1* is necessary for early specification of retromammillary progenitors destined to become STN. However, there are differences between mice and humans that remain to be explored, as *Nkx2-1* is downregulated after E12.5 in mouse postmitotic STN neurons, while in humans some STN neurons remain *Nkx2-1*^+^.

#### Foxa1/Foxa2

*Foxa1* [forkhead box A1 (hepatocyte nuclear factor 3α)] and *Foxa2* [forkhead box A2 (hepatocyte nuclear factor 3β)] belong to the vertebrate forkhead box family of TFs and have partially overlapping functions during embryonic development (Friedman and Kaestner, 2006; Kaestner, 2010). In the developing neural tube, *Foxa1* and *Foxa2* have been described in the notochord and in the floor plate at approximately E8.0 to E8.5, with *Foxa1* having weaker expression than *Foxa2* (Fig. 3; Ang et al., 1993; Mavromatakis et al., 2011; Puelles et al., 2012; Díaz et al., 2014). In the floor plate, they are a part of a transcriptional network that guides the development of dopaminergic mesodiencephalic progenitors. Both of these TFs have complex interactions with *Shh* (Mavromatakis et al., 2011). The best-studied role of *Foxa1* is its role in proliferation, specification, and maintenance of dopaminergic neurons, which is exhibited through complex interactions with other TFs like *Lmx1a* and *Lmx1b* (Ferri et al., 2007; Lin et al., 2009; Pristerà et al., 2015).

*Foxa1* expression was examined in the mouse hypothalamus where it was observed in the STN, supramammillary nucleus, ventral premammillary nucleus, VTA, and posterior hypothalamic area (Fig. 2, Table 1; Gasser et al., 2016). In a series of elegant experiments, Gasser et al. (2016) have proven the essential role of *Foxa1* in the development of STN. In wild-type mice, *Foxa1* expression in the developing STN was detected at E12.5 (Fig. 3). In the absence of *Foxa1*, STN neurons were born, but they did not differentiate and migrate dorsolaterally, a phenotype also observed in *Pitx2*-deficient mice. Moreover, *Foxa1*-deficient mice at birth had no PITX2, FOXP2, calretinin, and neurotensin expression at the level of presumptive STN. Postmitotic loss of *Foxa1* function did not affect the formation of STN, as evidenced by maintained FOXP2 and 5-HT2c receptor expression, but these mice displayed locomotion deficits, neurodegeneration, and cell loss in the STN (Gasser et al., 2016). Recent findings have described a specific subtype of neurons, Foxa1^+^/Nr4a2^–^, which originates in the caudal part of retromammillary area and migrates tangentially to become STN (López-González et al., 2021). On the other hand, FOXA2 was not expressed at any analyzed age [E14.5 and postnatal day 0 (P0)] in mouse STN, so the authors assumed that their functions in the developing STN are not overlapping (Gasser et al., 2016). However, Nouri and Awatramani (2017) found a specific subpopulation of *Dbx1*^+^ cells in the anterior part of the mesodiencephalic floor plate, which coexpresses PITX2, LMX1A, and FOXA2 in the developing and adult (P28) STN, supramammillary nucleus, and premammillary nuclei.

To conclude, *Foxa1* expression in progenitors is needed to establish STN phenotype, while the conflicting reports about the *Foxa2* expression in the STN indicate that this is a topic that needs further clarification. Based on these findings, *Foxa1*/*2* probably do not have overlapping functions in the STN.

#### LIM homeobox transcription factor 1α and LIM homeobox transcription factor 1β (Lmx1a/Lmx1b)

*Lmx1a/b* are members of the LIM homeobox family of transcription factors that are expressed during early embryogenesis. *Lmx1b* expression starts at approximately E7.5, whereas *Lmx1a* is expressed at approximately E8.5 to E9 (Doucet-Beaupré et al., 2015). In the CNS, its expression is necessary for the activity of the isthmic organizer, the differentiation of hindbrain serotonergic and spinal dorsal horn neurons (Asbreuk et al., 2002; Dai et al., 2008; Zou et al., 2009). The *Lmx1a* has been described as a dorsalizing agent and controls the development of the roof plate and its derivatives (Manzanares et al., 2000; Millonig et al., 2000). Nevertheless, both TFs are expressed in the ventral mesodiencephalic floor plate where they specify dopaminergic progenitors (Fig. 2*A*, Table 1; Lin et al., 2009; Deng et al., 2011; Doucet-Beaupré et al., 2015).

High levels of *Lmx1a* mRNA have been found in the mouse posterior hypothalamus, supramammillary and ventral premammillary nucleus, STN, VTA, and SNc from the embryonic period (E12.5) to the early postnatal period (P0–P7; Figs. 2*B*, 3; Failli et al., 2002; Zou et al., 2009; Hoekstra et al., 2013). When analyzing the expression pattern of these TFs in adult brain, there are conflicting reports. While some authors state there is no *Lmx1a* mRNA after P14 in the aforementioned regions (Hoekstra et al., 2013), other authors described sustained, yet weaker expression in adult animals at 6 months of age (Zou et al., 2009). Interestingly, double-labeling experiments revealed the almost overlapping expression pattern of *Lmx1a* mRNA and LMX1B protein in the STN (Zou et al., 2009).

*Lmx1b* expression was similarly analyzed in the postnatal mouse brain. These experiments showed sustained *Lmx1b* expression at all analyzed ages (from E12.5 to P14) in the posterior and lateral hypothalamus, STN and parasubthalamic nucleus (a small nucleus located at the medial border of rodent STN), and ventral premammillary nucleus (Fig. 2*B*, Table 1; Asbreuk et al., 2002; Dai et al., 2008). Nevertheless, the *in situ* hybridization (ISH) signal weakened after P14 (Dai et al., 2008). In these regions, *Lmx1b* was expressed in excitatory neurons, while in the SNc and raphe nuclei *Lmx1b*^+^ cells colocalized with dopaminergic and serotonergic cells, respectively (Dai et al., 2008). Another TF, PITX2, is necessary for the onset of LMX1B expression in the STN, with both of these TFs colocalizing in the embryonic and adult STN (Asbreuk et al., 2002; Skidmore et al., 2008).

In addition, it is possible that joint cooperative interactions between *Foxa1* and *Lmx1a/b* are required for the specification of STN neurons (Gasser et al., 2016). Moreover, *Lmx1a* may have two expression domains—one caudal, which gives rise to dopaminergic lineage, and the other rostral, in which *Lmx1a* expression overlaps with *Pitx2* and BarH-like homeobox 1 (*Barhl1*), giving rise to glutamatergic hypothalamic neurons and the prospective STN (Kee et al., 2017; Kim et al., 2020). Surprisingly, single-nucleus RNA-seq of *Pitx2-Cre*^+^ cells derived from adult (P28) mouse STN detected hardly any *Lmx1b*, yet *Lmx1a*, *Foxa1*, *Foxp1*, *Foxp2*, and *Barhl1* were abundantly expressed in all analyzed clusters (Wallén-Mackenzie et al., 2020).

In summary, *Lmx1a/b* are important factors in the specification of glutamatergic, dopaminergic, and serotonergic neurons in the hypothalamus, STN, and mesencephalon. Reports indicate that both genes are also present in adult neurons of the aforementioned structures. In the STN, *Lmx1a/b* need additional genes (e.g., *Pitx2* or *Barhl1*) to specify neuronal progenitors to correct phenotype.

### Transcription factors expressed in postmitotic neurons

#### Paired-like homeodomain 2

*Pitx2* belongs to the paired-like homeodomain family of TFs that is important for brain development (Muccielli et al., 1996; Smidt et al., 2000; Lamba et al., 2008; Waite et al., 2013). During development, *Pitx2* mRNA becomes detectable at E9.5 in postmitotic cells of the basal plate at the level of mesencephalic flexure (Muccielli et al., 1996). At later stages of development, from E10.5 to E13.5, *Pitx2* mRNA respects prosomeric boundaries and can be detected in two zones (Figs. 2*A*, 3). The first expression zone spans across the mesencephalon to the mammillary region, including the ZLI, ZI, retromammillary and mammillary areas, and basal plates of prosomeres p1–p3, with the second zone of expression being the alar plate of the mesencephalon (the primordium of the superior colliculi; Fig. 2, Table 1; Muccielli et al., 1996; Martin et al., 2002, 2004). From E16.5 throughout adulthood, *Pitx2* expression is prominent in the STN, ZI, the nuclei of mammillary complex, deep gray layer of the superior colliculus, and the anterior and intermediate lobes of the pituitary gland, indicating it has a role in the maintenance of neuronal identity (Martin et al., 2002, 2004; Waite and Martin, 2015; Xie and Dorsky, 2017; Guo and Li, 2019; Kim et al., 2020; Mickelsen et al., 2020).

The role of *Pitx2* in the development of the rodent STN has been thoroughly studied. Double-labeling immunohistochemical experiments demonstrated that PITX2*^+^* cells in the STN are neurons as they colocalize with NeuN and calretinin (Martin et al., 2002, 2004). However, *Pitx2* is not a determinant of a neurotransmitter phenotype, as it is expressed in both GABAergic cells of ventral mesencephalon (which will later become a part of the superior colliculus), and glutamatergic cells in the STN, posterior hypothalamus, and the mamillary region (Westmoreland et al., 2001; Martin et al., 2002; Schweizer et al., 2014; Waite and Martin, 2015). In wild-type mice, the migration of *Pitx2*^+^ cells is observed from E10.5 to E14.5, with the majority of migration taking place from E12.5 to E14.5, rostrally and laterodorsally from the retromammillary area to their position in the adult STN (Martin et al., 2004; Skidmore et al., 2008). In the *Pitx2* knock-out mice, no *Pitx2* mRNA can be observed at the level of the presumptive STN at E14.5, but the signal can be observed more medially, suggesting an arrested migration of future STN neurons (Martin et al., 2004; Skidmore et al., 2008; 2012). The studies characterizing subtypes of STN neurons showed that the expression of *Pitx2* is necessary for the expression of several key TFs in the STN. For example, the expression of *Pitx2* is necessary for the expression of *Lmx1b* in the STN, whereas the loss of *Lmx1b* does not preclude *Pitx2* expression (Asbreuk et al., 2002; Skidmore et al., 2008). The colocalization of *Pitx2* with *Lmx1b* in the STN persists postnatally (Asbreuk et al., 2002). On the other hand, the expression of FOXP1 and FOXP2 was preserved, but reduced in the murine embryonic STN lacking *Pitx2* function (Skidmore et al., 2008), suggesting that other neuronal lineages beside *Pitx2^+^* exist in the STN. It has been suggested that *Pitx2*, along with *Barhl1*, could be used as markers of a specific subset of *Lmx1a*^+^ progenitors adapting a rostral, glutamatergic fate in the ventral mesodiencephalic area, thus distinguishing developing STN neurons from dopaminergic neurons (Kee et al., 2017).

*Pitx2* is now an established marker of postmitotic STN neurons; therefore, *Pitx2* promotor is now widely used in various experimental settings when precise labeling of STN neurons is required, for example, single-cell/nuclei sequencing or creating conditional transgenic mice (Schweizer et al., 2014, 2016; Wallén-Mackenzie et al., 2020). However, one must be careful when interpreting data from these studies as not all STN neurons are *Pitx2*^+^ (Skidmore et al., 2008).

#### BarH-like homeobox 1

*Barhl1* is a member of the BarH gene family, which has a restricted expression in the CNS during development (Bulfone et al., 2000; Reig et al., 2007). *Barhl1* expression was first detected in the caudal diencephalon at E9.5. At E10.5, the rostral expression domain encompasses the basal plate of p1–p3 and hp1, the ZLI, and the pretectum (p1), whereas the caudal expression domain is confined to the midbrain–hindbrain boundary (Figs. 2*A*, 3; Bulfone et al., 2000; Rachidi and Lopes, 2006). Interestingly, from E12.5 to birth, *Barhl1* is expressed in the mammillary region and in the alar plate forming the inferior and superior colliculi, similar to the expression pattern reported for *Pitx2* (Fig. 2*B*, Table 1). In the hindbrain, *Barhl1* may be found in the rhombic lips, a transient structure giving rise to cerebellar granular cells (Bulfone et al., 2000; Lopes et al., 2006; Rachidi and Lopes, 2006). Importantly, these expression patterns have also been observed in human embryonic and fetal brain (Lopes et al., 2006). There are indications that *Barhl1* expression, at least in the basal plate, is dependent on *Shh* signaling, while its alar expression is regulated by BMPs (Bulfone et al., 2000; Lopes et al., 2006; Rachidi and Lopes, 2006; Martinez-Lopez et al., 2015).

*Barhl1* expression in STN was specifically described in adult (P28) *Pitx2-Cre* and embryonic *Lmx1a*^EGFP^ mice, leading to a conclusion that there is a significant proportion of *Pitx2-Barhl1* or *Lmx1a-Barhl1* colocalization in STN cells, with high levels of expression and significant overlap of all three TFs in the adult STN (Kee et al., 2017; Wallén-Mackenzie et al., 2020).

#### Forkhead box 1 and Forkhead box 2

Forkhead box 1 (*Foxp1*) and *Foxp2*, members of the forkhead box family of TFs, are best known for their role in brain development and acquiring speech and language functions (Enard et al., 2002). In the developing murine brain, *Foxp1* and *Foxp2* are expressed after E12.5 and their expression is sustained during adulthood, although at lower levels (Ferland et al., 2003; Takahashi et al., 2003; Co et al., 2020). *Foxp1/2* hypothalamic expression was limited to the basal plate of hp1 prosomere, specifically the supramammillary, ventral premammillary, and mammillary nuclei (Fig. 2*A*, Table 1; Ferland et al., 2003; Teramitsu et al., 2004; Kim et al., 2020; Mickelsen et al., 2020).

In the developing mouse STN, *Foxp1* mRNA was first observed at E13.5 and persisted until P14 (Fig. 3). Interestingly, adult mouse STN apparently has no *Foxp1* mRNA signal (Tamura et al., 2004; Philips et al., 2005). In the developing human brain, both *FOXP1* and *FOXP2* were expressed in the STN during midgestation (22 postconceptional weeks); however, *FOXP2* expression was stronger (Teramitsu et al., 2004), and the FOXP2 protein can be detected in adult STN (Bokulić et al., 2021). These postmitotic markers were found in human fetal striatum at 11 gestational weeks, so perhaps they are also expressed in the STN earlier than previously reported (Pauly et al., 2013). As was already discussed, there have been attempts at profiling subtypes of subthalamic neurons. Despite *Pitx2* being widely used as a marker of STN neurons, some results suggest there is a subtype of *Pitx2*-/*Foxp1^+^* or *Foxp2^+^* neurons (Skidmore et al., 2008). Single-cell RNA sequencing of mouse ventral mesodiencephalic area assigned *Foxp1* and *Foxp2* to both developing glutamatergic and dopaminergic populations (Kee et al., 2017).

*Foxp1*/*2* are also important for the specification of various neuronal populations in the basal ganglia. *Foxp1* and *Foxp2* mRNA appears in the subventricular zone of the LGE. Both TFs are expressed in postmigratory striatal projection neurons, with *Foxp2* mRNA in striosomes and *Foxp1* mRNA equally expressed in matrix and striosomes (Takahashi et al., 2003; Tamura et al., 2004; Teramitsu et al., 2004; Takahashi et al., 2010; Fong et al., 2018; Co et al., 2020). Interestingly, *Foxp1*/*2* expression patterns diverge in other basal ganglia structures. *Foxp1* mRNA is absent from both mouse and human GP, whereas *Foxp2* is expressed at low levels (Ferland et al., 2003; Tamura et al., 2004; Co et al., 2020). The SN has both *Foxp1* and *Foxp2* expression, although *Foxp2* is more prominent (Fig. 2*B*, Table 1; Ferland et al., 2003). *Foxp2* is now an established marker of a specific, arkypallidal neuronal population in the GPe, which originates from the LGE/CGE (caudal ganglionic eminence) and projects to the striatum (Abdi et al., 2015; Dodson et al., 2015).

In conclusion, *Foxp1*/*2* are important determinants of basal ganglia neuronal populations and hypothalamic nuclei derived from the basal plate of the prosomere hp1. Both of these genes can be found in the STN where they possibly determine a *Pitx2*-independent neuronal subpopulation, but this remains to be further explored with double-labeling experiments.

### The transcriptional profile of subthalamic nucleus

Studies using the classical model of diencephalic development generated many important pieces of data about the place of origin of STNs. However, the advent of molecular biology exposed all the shortcomings of classical anatomic/morphologic concept. A conceptual framework of the prosomeric model gave us a full appreciation of the vast data on gene expression in the developing hypothalamic and diencephalic primordium. Within this framework, it became easier to trace the lineage of individual neurons and nuclei. Analysis of transcription factors involved in the development of the STN and surrounding structures taught us many important lessons.

First, it appears that there is no single specific marker of STN neurons. The diversity of TFs found in the STN suggests that this nucleus harbors a more transcriptionally heterogeneous neuronal population than was previously thought. This finding is not surprising given that single-cell sequencing experiments revealed a complex composition of other basal hypothalamic nuclei (i.e., mammillary bodies, supramammillary, and ventral premammillary nuclei; Mickelsen et al., 2020; López-González et al., 2021). Indeed, if we summarize our findings, we find that there is a set of shared TFs among the STN, surrounding hypothalamic glutamatergic neurons, and mesodiencephalic dopaminergic neurons. The neuronal population of the STN is probably specified by a combination of the discussed TFs and depends on their timing and level of expression. For instance, *Foxa1* and *Lmx1a/b* can be found in the mesodiencephalic dopaminergic neurons and in the basal hypothalamus (Kee et al., 2017; Kim et al., 2020; Mickelsen et al., 2020; Zhang et al., 2021). The expression of these TFs points to a shared progenitor zone in the floor/basal plate, which is probably influenced by *Shh* signaling. However, as discussed by Nouri and Awatramani (2017), *Dbx1* could indicate an anterior floor plate region that will eventually give rise to glutamatergic populations like the STN. Taking into account everything discussed thus far, one can hypothesize that the progenitors of the glutamatergic STN could be defined by the expression of *Dbx1*, *Foxa1*, *and Lmx1a/b* (Fig. 3). As these progenitors exit cell cycle, they start to express postmitotic markers *Barhl1*, *Foxp1/2*, and *Pitx2* (Fig. 3; Kee et al., 2017; Kim et al., 2020; Mickelsen et al., 2020; Zhang et al., 2021). The exact spatiotemporal expression of these TFs in STN remains to be fully elucidated. Also, *Nkx2-1* is clearly indispensable for the formation of the basal hypothalamus in rodent models, yet it is absent from the STN from E12.5 onward (Kimura et al., 1996; Nakamura et al., 2001; Kim et al., 2020; Murcia-Ramón et al., 2020). Nevertheless, there have been reports of NKX2-1 expression in the adult human STN (Bokulić et al., 2021), so these discrepancies between model animals and humans remain to be further investigated.

Contrary to molecular phenotype, connectivity and functional properties of STN have been extensively studied in animal models, human and primate neuroimaging, and electrophysiological experiments (Mallet et al., 2007; Lambert et al., 2012; Haynes and Haber, 2013; Alkemade et al., 2015). According to the prevailing tripartite anatomofunctional division of the STN, the dorsal part processes sensorimotor information, while the ventral part processes cognitive and affective information (Alexander et al., 1990; Parent and Hazrati, 1995; Joel and Weiner, 1997; Temel et al., 2005). The data about the molecular phenotype of neuronal subpopulations in these functional regions is still lacking. Progress has been made in this field with the work of Parolari et al. (2021), who showed that a subpopulation of STN neurons expressing GABA_A_ receptor subunit *Gabrr3* projects to the GP and SNr and modulates repetitive grooming in mice. Further studies are needed to link molecularly distinct subpopulations with the observed patterns of connectivity and functional properties.

## Clinical Importance of STN

The involvement of STN in motor control has been demonstrated first in patients with hemiballism, a neurologic disorder that manifests with unilateral, involuntary spasmodic movements of limbs (Postuma and Lang, 2003; Marani et al., 2008). Nowadays, STN is clinically important as a deep-brain stimulation (DBS) target for the treatment of PD (Kumar et al., 1998; Hamani et al., 2005; Benabid et al., 2009). However, the STN is not purely a motor nucleus. Several studies in experimental animals demonstrated that the STN modulates different aspects of behavior such as repetitive and compulsive behavior (Winter et al., 2008; Lewis et al., 2018; Parolari et al., 2021), motivational processes (Rouaud et al., 2010; Pelloux and Baunez, 2013), and impulsivity and inhibition control (Baunez and Lardeux, 2011; Rossi et al., 2015). The quest to elucidate the functional roles of STN in the human brain is limited to observing the side effects of DBS in parkinsonian patients (Jahanshahi et al., 2000; Temel et al., 2005; Voon et al., 2006; Saleh and Okun, 2008; Voon et al., 2017). Another possible avenue of human STN research is the use of NDDs as “naturally occurring experiments.” Because of the disruption of normal brain development in NDDs, one can link the observed behavioral changes to the changes in gene expression, number, and morphology of neurons or neuronal connectivity patterns. Currently, there is no evidence for the involvement of STN in any NDD, primarily because of the lack of studies investigating this question. However, there are indirect evidence that STN might be involved in some NDDs.

In a current model of NDDs, a genetic mutation or a disruption of a developmental process leads to structural changes in specific brain regions, which lead to functional and behavioral changes (Mitchell, 2015). The genes involved in the pathophysiology of NDDs code for a vast array of proteins (Mitchell, 2011), but here we will describe morphogens (e.g., *SHH* and *FGF8*; Dubourg et al., 2007; Roessler and Muenke, 2010; Dubourg et al., 2016; Roessler et al., 2018) and transcription factors (e.g., *FOXP1* and *FOXP2*; Enard, 2011; Bacon and Rappold, 2012; Meerschaut et al., 2017; Braden et al., 2021). Mutations in morphogenes can cause vast developmental defects, whereas mutations in TFs lead to more subtle phenotypes. Holoprosencephaly (HPE) is a multietiological NDD that serves as an example of an NDD where a disruption of a basic neurodevelopmental plan leads to observable brain and craniofacial malformations (ten Donkelaar et al., 2014b). Mutations of the *SHH* signaling pathway and *FGF8* gene are commonly found in patients with HPE (Dubourg et al., 2016; Roessler et al., 2018). In a chapter about induction and patterning of the forebrain, we discussed the complex spatiotemporal expression patterns of these morphogens, which could account for the great variability of the clinical phenotypes of HPE (ten Donkelaar et al., 2014b; Diaz and Puelles, 2020). On the other hand, cognitive impairments caused by mutations in *FOXP1* and *FOXP2* genes are perceived as neurobehavioral disorders, or NDDs in a “broader sense” (ten Donkelaar et al., 2014a). Neurobehavioral disorders like autism spectrum disorder (ASD), schizophrenia, intellectual disability, and attention deficit-hyperactivity disorder (ADHD) are characterized by the presence of various neuropsychiatric symptoms (ten Donkelaar et al., 2014a; Mitchell, 2015). The etiology of such disorders is complex, however much effort is put into elucidating genetic causes (Mitchell, 2011). Although *FOXP1* and *FOXP2* have a high degree of sequence similarity, their mutations cause different NDDs. Mutations in *FOXP1* gene result in a complex, global NDD with behavioral disorders, brain malformations, specific facial features, and malformations of other organ systems (Co et al., 2020). This prompted clinicians to define a separate NDD, *FOXP1-*related intellectual disability syndrome (Meerschaut et al., 2017; Lozano et al., 2021). Conversely, *FOXP2* mutations lead to severe speech and language impairments, but these can be attributed to impaired brain connectivity, not to overt structural brain abnormalities (Lai et al., 2003; Co et al., 2020).

It is interesting to note that some of the genes linked to NDDs are also essential for the development of the STN (Table 2). When interpreting these findings, one has to be careful to acknowledge that neurobehavioral disorders have complex etiologies that cannot be limited to one gene. Similarly, mutations in these genes probably affect a wide repertoire of downstream targets, and their effect depends on how they affect the final protein product (Parenti et al., 2020). If we examine clinical manifestations of several NDDs, some symptoms point to a dysfunction of cortical-basal ganglia circuits (Riva et al., 2018; Kuo and Liu, 2019; Vicente et al., 2020). In addition to locomotor control, the basal ganglia have important roles in nonmotor functions like executive functions, procedural learning, habit formation, and goal-directed behavior (Graybiel, 2008; Leisman and Melillo, 2013; Haber, 2016). These functions were mostly attributed to frontostriatal connections, so striatal dysfunction is investigated in the pathophysiology of NDDs like ASD, ADHD, and schizophrenia (Leisman and Melillo, 2013; Kuo and Liu, 2019; Vicente et al., 2020). The STN can be perceived as another input nucleus of the cortical-basal ganglia circuits because it receives excitatory cortical input via the hyperdirect pathway (Nambu et al., 2002; Baunez and Lardeux, 2011; Tewari et al., 2016). The hyperdirect pathway has an important role in nonmotor functions of STN, like decision-making, attention, action control, and motivated behavior (Baunez and Lardeux, 2011; Weintraub and Zaghloul, 2013; Aron et al., 2016; Bonnevie and Zaghloul, 2019). Furthermore, the recently proposed hypothalamic origin of STN corroborates the complementary roles of hypothalamic and basal ganglia circuitry in motivated behavior. In this model, the STN serves as a “stop signal,” pausing initiated behavior (Barbier and Risold, 2021). Based on the discussed motor and nonmotor functions of the STN, especially its role in action inhibition, we argue that it probably plays an important, yet underexplored role in NDDs. For example, ASD, ADHD, Tourette’s syndrome, and schizophrenia are all characterized by restricted and repetitive patterns of behavior (RRBs) like stereotypies, perseveration, and tics, which are basically disorders of behavioral control (Lewis et al., 2018; Vicente et al., 2020; Tian et al., 2022). Additionally, RRBs could be just behavioral manifestations of a broader cognitive inflexibility and an inability to stop unwanted actions or thoughts (Tian et al., 2022). However, clinical and preclinical neuroimaging studies have not studied intra-basal ganglia connectivity or possible abnormalities of small nuclei like STN in RRBs (for review, see Wilkes and Lewis, 2018), although animal models demonstrated clear involvement of STN in stereotyped behavior (Chang et al., 2016; Lewis et al., 2018; Wilkes and Lewis, 2018). Genes implicated in speech impairment (*FOXP1* and *FOXP2*) have widespread expression in the basal ganglia, STN included (Enard, 2011; Bacon and Rappold, 2012; Co et al., 2020; Bokulić et al., 2021). These expression patterns, coupled with the role of basal ganglia in language acquisition (Eigsti et al., 2011; Enard, 2011) and speech impairment being recognized as a side effect of DBS (Pützer et al., 2008; Silveri et al., 2012; Ehlen et al., 2014), point to a possible involvement of STN in speech production. Neuroimaging studies could give us valuable insight into the role of STN in speech production by exploring possible differences in STN connectivity and volumes in healthy control subjects and people with language impairments, whereas human postmortem studies could analyze the transcriptional profile of STN in healthy and language-impaired individuals.

**Table 2:** A summary of morphogens and transcription factors implicated in the development of STN that have previously been linked to various neurodevelopmental disorders

Morphogen/ Transcription factor	Neurodevelopmental disorder	Reference
*SHH*	Holoprosencephaly	Dubourg et al., 2007; Roessler and Muenke, 2010
	Schizophrenia	Betcheva et al., 2013; Boyd et al., 2015; Yao et al., 2016
	Autism spectrum disorder	Yao et al., 2016; Patel et al., 2017; Kumar et al., 2019; Upadhyay et al., 2021
	Attention deficit-hyperactivity disorder	Heussler et al., 2002
	Language impairment	Santiago et al., 2006
*FGF8*	Holoprosencephaly	Dubourg et al., 2016; Roessler et al., 2018
	Autism spectrum disorder	Clarke et al., 2012; Rubenstein, 2010
*NKX2-1*	Schizophrenia	Malt et al., 2016
	Autism spectrum disorder (in BHC)	Milone et al., 2019
	Attention deficit-hyperactivity disorder (in BHC)	Gras et al., 2012
*LMX1A/LMX1B*	Schizophrenia	Bergman et al., 2010
	Autism spectrum disorder (*LMX1B*)	Thanseem et al., 2011
*FOXP1/FOXP2*	Autism spectrum disorder	Bacon and Rappold, 2012; Cristino et al., 2014; Co et al., 2020
	Schizophrenia (*FOXP2*)	Tolosa et al., 2010
	Speech and language disorder	Enard et al., 2002; Lai et al., 2003
	*FOXP1*-related intellectual disability syndrome	Meerschaut et al., 2017; Lozano et al., 2021

BHC, Benign hereditary chorea.

In summary, a shared set of symptoms among NDDs might suggest a common cortical-basal ganglia-thalamocortical dysfunction. The exact role that the STN has in the pathophysiology of RRBs and impaired verbal fluency is still poorly understood, but we can hypothesize that the imbalance between the direct and indirect pathways and the loss of inhibitory control of STN are key components. Before exploring the functional roles of STN in NDDs, we should have an understanding of its cellular and molecular properties. The analysis of STN in NDDs could provide us with valuable information about the cellular composition of STN.

## Conclusion

In a quest to elucidate the developmental origin and molecular profile of STN neurons, a powerful tool at our disposal is genetically modified experimental animals. These models provided us abundant data about rodent STN. The analysis of gene expression patterns and lineage tracing experiments have proven the hypothalamic origin of STN. However, the origin and the transcriptional profile of the human STN remain to be further investigated, as there appear to be some interspecies differences. When analyzing the development of the human STN such tools are not available, and the majority of studies are descriptive studies using postmortem materials.

The literature search for studies of human STN revealed there are hardly any studies that analyzed the molecular phenotype or developmental origin of STN neurons. The majority of studies dealing with human STN analyzed expression patterns of calcium-binding proteins and neurotransmitter receptors in STN (Lévesque and Parent, 2005; Zwirner et al., 2017; Wu et al., 2018; Alkemade et al., 2019), with only one study also analyzing TF expression (Bokulić et al., 2021). However, all these studies analyzed the adult STN, so the data about TFs in the developing human STN is lacking. Future human postmortem studies should focus on comparing the expression patterns of TFs in rodent and human developing STNs, thus giving us insight into possible species-specific differences in cellular composition or developmental origin. Moreover, thorough profiling of the neuronal populations of STN with TFs, calcium-binding proteins, and membrane receptors would allow modifications of existing models of cytoarchitectonic organization.

This comprehensive summary of TFs expressed in the STN sets the ground for future human postmortem studies. These studies should comparatively analyze the development and expression patterns of NDD-related genes in the STN between neurotypical and neurodivergent individuals. The results of these studies would enhance our understanding of the pathophysiology and symptomatology of NDDs.

## Synthesis

Reviewing Editor: Christopher Colwell, UCLA School of Medicine Decisions are customarily a result of the Reviewing Editor and the peer reviewers coming together and discussing their recommendations until a consensus is reached. When revisions are invited, a fact-based synthesis statement explaining their decision and outlining what is needed to prepare a revision will be listed below. The following reviewer(s) agreed to reveal their identity: NONE.

Synthesis: Both reviewers feel that the authors have done a nice job summarizing extensive data in the field on the developmental origins of STN and the associated gene expression profiles.

Major issues raised by the reviewers include:

(1) Claims made in section 6 regarding connections between STN and neurodevelopmental disorders are a bit too broad - for instance, the assumption that shared genetic causes, and thus shared developmental brain dysfunction can be representations of a common underlying mechanism does not have sufficient supporting evidence; mutations in developmental genes likely have a wide repertoire of downstream effects dependent upon how it affects the gene product; similarly, shared symptoms does not imply shared underlying etiologies.

(2) Recommend authors explore Allen Brain Atlas’ human developmental in situ hybridizations which have at least some of the genes discussed here (e.g. Nkx2.1) which could aid in further clarification of its human expression profile (ABA also has a non-human primate catalog which may be more extensive)

3) The article is verbose, introduction parts can be reduced (see below).

4) It remains unclear why especially these transcriptional factors have been chosen/studied for review. This should be clarified.

5) Since this is a review, some of the experimental animals used by others should be named: human and other mammal results can strongly differ as Authors indicate themselves.

Detailed comments:

r4: stating that the STN is a glutamatergic nucleus only holds for its projections to globus pallidus int. and substantia nigra pars compacta, e.g. 95% of the STN neurons are NO synthase positive (Nisbet Brain Res Mol Brain Res 22: 329-332).

r35-37: the position of the STN varies in mammals: the STN in humans is dorso-lateral to SN, dorsal of the capsula interna, lateral of the red nucleus and lateral of the lateral hypothalamic area rostral (see Mai et al, 1997, Atlas of the human brain, AP), and in the rat its position is different. Au’s could describe the human position especially in respect with part 6 (see below).

r42 the cellular and cytological composition of the STN have been researched for rat, guinea-pig, cat, pig, monkey humans. It concerns: amount of cells and its cell types, types of boutons, types of vesicles, presence of interneurons (e.g. Romansky and Usunoff, 1978 Etud Balk 31:1225-1228, 1980 J Hirnforsch 21:515-521 and Anat Embryol 159: 163-180, 1983 Neurosci Lett 42:113-117, 1985 J Hirnforsch 26:259-273, 1987 J Hirnforsch 28:407-433). Cell types, also in relation to connections, have been published for humans and several other experimental animals, to name only a few: Kitai et al, 1983 J Comp Neurol 215: 245-257, rat; the Parent group e.g. Brain Res 436: 296-310 and Brain Res Bull 10: 529-537, humans, Iwahori 1978 J Comp Neurol 182: 383-397, cat.

r56-99: first some history. The neuromeric pattern has been described for the first time by Orr (1887, J Morphol 1: 311-372). W. His contributed by the description of the sulcus limitans (Hiss 1888 Abh k Sächs Ges Wiss Math-Phys Kl 14: 341-372 and in 1890 part 15: 675-735). The pro-neuromeres were already described by Berquist and Källén, see 1954 J Comp Neurol 100: 627-660 and see Tsai et al. 1981, parts I and II, J Comp Neurol 198: 275-306. By the way what is the difference between pro-neuromeres and prosomeres? It is sufficient to indicate in this part that the neuromeric pattern is unclear in pros- and mesencephalon. The conflicts in literature concern the sulcus limitans, number of neuromeres and the localization the sensory-motor columns in the prosencephalon (best citation presumably Gribnau and Geijsberts Adv Anat Embryol Cell Biol 91: 1-63). An extra problem was that the proneuromeres disappeared before the neuromeres appeared. By this time laps the two phenomena could hardly be connected. It is then the place to introduce Rubenstein’s prosomeric model.

Authors could consider to import in their article the E10.5 and/or E12.5 figure of Rubenstein published in Science 266, 1994. It makes it clear to non-hypothalamic researchers.

r95-99 could be reduced Author’s should check whether all citations are needed or not.

r100-116: stating that borders are present and the hypothalamus can be divided in two prosomeres, without showing the borders in an embryo makes it difficult for the reader to understand the text. This is an extra argument to import figure(s) of Rubenstein. Moreover, the text presumably can be reduced due to the extra figure(s).

r118-175: this referee leaves to Au’s stating that the older literature had a vague definition of the subthalamic region. Nevertheless, in the whole paragraph the autoradiographic study of Keyser in the Chinese hamster is missing. This study should have been essential for Au’s. Keyser’s thesis (1972) describes the diencephalon development and in the Handbook of the hypothalamus the development of the hypothalamus has been treated and clear STN results are given:

Keyser A (1972), The development of the diencephalon of the Chinese hamster. An investigation of the validity of the criteria of subdivision of the brain. Thesis Nijmegen, The Netherlands (also publ. in Acta Anat (Basel) 83 (suppl 59: 1-178

Keyser A (1979) Development of the hypothalamus in mammals. An investigation into its morphological position during ontogenesis. In Morgan PJ, Panksepp J (eds.) Handbook of the hypothalamus vol I. Marcel Dekker Inc. NY, Basel pp65-136

This referee steals text from Marani et al., The subthalamic nucleus, Part 1. Adv Anat Embryol Cell Biol 198, to give a summary of Keyser’s work, so Au’s can decide (with excuses for typing errors in advance): The regio subthalamica in the Chinese hamster develops rostral of the synencephalon (future regio pretectalis) and basal to both the pars dorsalis (parencephalon posterior) and ventralis thalami (parencephalon anterior) (for localization of these parts on the ventricular surface see Fig. 7 ). At E 12 ( embryonic day 12) the neuromeres are clearly discernable. They show that the basal parts of the future regio pretectalis, the area below it (the future regio subthalamica), and the area caudal to the eyestalk are advanced in development compared to the other ventricular areas. At E 13 a mantle layer can be discerned in these areas. A compact “stream” (Keyser 1972) of cells can be noted and is called the subthalamic cell cord. This cell cord does not contain condensations or a development that indicates pronuclei. The cord reaches from its basal part below the future main thalamic (parencephalic) areas towards the diencephalic telencephalic border that can be recognized by the external telodiencephalic groove (also called the sulcus haemisphericus). So, early in development (E 12 -E 13 ) a subthalamic region can be discerned behind the mammillary recessus and a subthalamic cell cord extending from this area, reaching to the telencephalic-diencephalic border. At E 14 the prerubral tegmentum contains the pronuclei of the nuclei of Cajal and Darkschewitsch and it continues without clear borders in the more rostrally situated regio subthalamica. The basal area of the subthalamic cell cord is slightly more developed than the rest of this cell cord. It is this stage of development in which the growth of the mantle layer obscures the neuromeric borders. However, the neuromeric subdivisions can still be discerned in the basal/caudal diencephalic part, delineating the subthalamic area and the start of its cell cord. It should be noted here that the basal regio subthalamica is sandwiched between the tegmentum prerubralis and the regio mamillaris, while from its rostral part the subthalamic cell cord rises towards the diencephalic-telencephalic border. At E 15, within the regio subthalamica develops the supramammillary commissure that crosses the midline. The development of the regio subthalamica is advanced, and it is proposed that this area is “an organizer centre for the rostralmost parts of the brain” (Keyser 1972). The corpus subthalamicum Luysii (the future nucleus subthalamicus) is recognizable at this stage. “It develops from a tangentially migrating stream of cells that, originating from the matrix of the supramammillary recess, gradually shifts in a rostrodorsal direction” (Keyser 1972). At E 16 the subthalamic cell cord has developed into the suprapeduncular complex and a superficial corpus subthalamicum can be recognized. The suprapeduncular complex contains several subdivisions, but seemingly contributes to the entopeduncular nucleus and the globus pallidus (the rostral extension of the suprapeduncular complex). Translated into human terminology, it contributes to the globus pallidus internus and externus, respectively. A posterior hypothalamic area is considered to develop in relation to the corpus subthalamicum. The future corpus Luysii has a latero-dorsal migration, while the remnants of the subthalamic cell cord contain a more medio-dorsal development, in fact separating both cell populations. Their base is already loosened at E 16 . Thus, the subthalamic area really undergoes a tangential migration, in which the corpus Lsii takes the most lateral route. The autoradiographic results in the Chinese hamster show that the area subthalamica is characterized by an early onset of differentiation. It is autoradiographically labelled from E 12 until E 18 onwards, being one of the first labelled areas. It originates from the medially located ventricular surface of the area above the mammillary recess and spreads tangentially. The most laterally situated cells of the corpus Luysii are born on E 13 to E 18 . The tangential migration is also supported by the autoradiographic results.

Au’s can disagree with these results, but at least they should have mentioned these results, since autoradiography is a strong, basic technique in embryology and indeed can follow developing neuronal cells to a large extend.

r177-193: It is still worthwhile to look into the review article of Claudio Stern, who summarized in 2001 the expectations of the future research: It also seems likely that more complex mechanisms, perhaps including some degree of pre-patterning (or differential competence) of the prospective neural plate, and/or a role for differential timing of exposure to organizer-derived signals will be revealed by future research. (Stern, 2001, Stern CD (2001) Initial pattering of the central nervous system: How many organizers. Nature Reviews Neurosci 2: 92-98). Moreover, in the description of concentration gradients of secreted morphogens should be added, according to this referee: e.g. Schmidt et al. See ref in: 2008 The role of Wnt signalling in the development of somites and neural crest, in Adv Anat Embryol Cell Biol 195:1- 64. There are more researchers that contributed to where we are now (Bronner-Fraser?).

r196-210: The underlying problem is when are meso-ectoderm cells deposited into the embryo by both the epiblast and ectoderm. See e.g. Vermeij-Keers and Poelmann, 1980 Neth J Zool 30: 74-81; Smits van Prooije et al., 1985, Anat Embryol 176: 71-77. A better worked out text could be made.

r195-270: SHH and Wnt do appear in various segments/ neuromeres/meso-ectoderm cells. The appearance of both morphogens don’t solely characterise distinct neuronal localisations (pure topography), but is also the consequences of general functional processes belonging to development and these processes are subject to time lapses within one and the same area. In other words, using these morphogens as being exclusively a local marker seems unwarranted, since these morphogens pass over neurons and neuronal networks in a wave-like manner or can be considered as an autonomous progression of differentiation. (see Chandebois and Faber, Automation in animal development, Monographs in Developmental Biology ed. Wolsky A, 16: 1-204). No argument can be borrowed from these morphogens to prove that the STN is hypothalamic (hp1 or hp2) in origin.

r293-296: Fg/8 is only postulated to play an important role in STN development, because Fg/8 is involved in the development of the retromammilary area(?): Clearly a postulate.

Parts 4.1 to 4.3 are weak arguments to place these morphogens in a clear relation to early STN-hypothalamic development.

r311-463 (5.1) contains a fine description of the transcription factors present in neural progenitors: remark on Dbx1, the arcuate nucleus contains dopaminergic cells and on Nkx2-1, the tangential movement has been described by Keyser for the first time.

r465-566 (5.2) good overview of the transcription factors in postmitotic neurons

r611-685 (6) On first reading this referee thought to skip part 6 till he realised that it is the construct of the text. Proposal: 1) show that deficiencies in morphogens or transcription factors lead to aberrations or deflections in organs or tissues (back ground), e.g. like in Clinical neuroembryology (Springer) by ten Donkelaar, Lammens and Hori. I suppose that the Au’s do have more and presumably better examples (plural!). 2) Take one of the disorder examples that have been well worked out (convincing argument) 3) summarize the other cases. It is a temptation to relate the early developmental morphogens and transcription factors to neurodevelopmental disorders and therefore the description should stay in.

Small errors or indistinctions that should be corrected:

r576: Marani, Subthalamic nucleus part 1 does not say that the STN is homogeneous. Look into part 2 (Heida, The subthalamic nucleus part II Modelling and simulation activity, Springer, 2008) at the end you will find the tuning of part 1 and 2 in which based on physiology two types of neurons are discerned and the presence of interneurons is described.

r581-583: “” there is a set of shared TFs 582 between the STN, mammillary, supramammillary, and ventral premammillary nuclei, and mesodiencephalic dopaminergic neurons"”, but what then determines the STN?

r587-588: Dbx1 presumably gives rise to glutamatergic populations. Fact or presupposition?

## Author Response

AUTHOR’S REBUTTAL - A POINT TO POINT REPLY TO REVIEWERS

We thank the reviewers and the reviewing editor for their helpful and thoughtful comments. We incorporated all the suggestions to the new revised manuscript. Our replies to the reviewers can be found in the following sections.

Major issues raised by the reviewers include:

(1) Claims made in section 6 regarding connections between STN and neurodevelopmental disorders are a bit too broad - for instance, the assumption that shared genetic causes, and thus shared developmental brain dysfunction can be representations of a common underlying mechanism does not have sufficient supporting evidence; mutations in developmental genes likely have a wide repertoire of downstream effects dependent upon how it affects the gene product; similarly, shared symptoms does not imply shared underlying etiologies.

We thank the reviewers for the comment. We agree that some of the assumptions made in paragraph 6 could have different explanations. However, the purpose of paragraph 6 was not to cover all possible causes of NDDs, their effects on brain development, and subsequent symptoms, but to shed some light on the possibility that the STN might have a role in the pathophysiology of NDDs. However, in order to make our intentions clear, we have modified the text in chapter 6 (see new chapter 6 and our response at the end of this file).

(2) Recommend authors explore Allen Brain Atlas’ human developmental in situ hybridizations which have at least some of the genes discussed here (e.g. Nkx2.1) which could aid in further clarification of its human expression profile (ABA also has a non-human primate catalog which may be more extensive)

The selected genes covered in the manuscript were curated from the available data in the literature on the development of STN and surrounding structures (e.g., globus pallidus, hypothalamus, thalamus, substantia nigra, etc.). We have also explored Allen Brain Atlas’ (ABA) mouse and human developmental in situ hybridizations and the data has been used to define transcription factors that are important for the development of STN. However, the analyzed human developmental ISHs did not have all of TFs of interest (for example, FOXA1, PITX2, LMX1A/B, DBX1 are missing), the ISHs of available TFs (NKX2-1, FOXP1, FOXP2) are often of poor quality (see for example FOXP2: https://www.brainspan.org/ish/gene/show/60634), and the expression was analyzed in other brain regions (e.g., amygdala, hippocampus, striatum). However, we investigated ABA’s human prenatal microarray data to extricate a list of differentially expressed genes. For example, we compared the STN to other structures and found PITX2, FOXA1, and BARHL1 among genes with the biggest fold change. Similar results were obtained when exploring human adult microarray data with search parameter STN vs.brain. Genes with the biggest fold change were again FOXA1, BARHL1, PITX2, with the addition of LMX1A and FOXP2.

We added appropriate text in the manuscript to make it clear how were genes presented in the manuscript selected.

3) The article is verbose, introduction parts can be reduced (see below).

We agree with the reviewers, thus we have appropriately shortened the introduction part. We also added suggested figure from the paper by Rubenstein (Science 266, 1994) and moved appropriate parts from the introduction to the figure description.

4) It remains unclear why especially these transcriptional factors have been chosen/studied for review. This should be clarified.

As we have already mentioned, we chose TFs based on the data from the literature, observed expression in the ABA’s mouse ISHs and human microarray data.

5) Since this is a review, some of the experimental animals used by others should be named: human and other mammal results can strongly differ as Authors indicate themselves.

We agree with the reviewers that data from other experimental animals is valuable and could differ from human data. However, when analyzing gene expression in the STN, we chose to primarily focus on mouse model. Due to the availability of genetically modified mice, mouse models gave the most data on STN development and gene expression. However, when appropriate data from other species (and especially human) was available, we incorporated it in our text and noted the species in which data was generated. Unfortunately, there are only scarce reports on TF expression in humans or other species. In the paragraph that describes the induction and patterning of the hypothalamic primordium, animal species were not sometimes mentioned because these developmental processes are similar in all vertebrates. However, if a mechanism is specific for mammals, we explicitly stated that

To make it clear, before the description of TFs in the developing STN (part 5), we emphasized that all data presented in the paragraph concerns mouse brain, if not specifically stated otherwise.

r4: stating that the STN is a glutamatergic nucleus only holds for its projections to globus pallidus int. and substantia nigra pars compacta, e.g. 95% of the STN neurons are NO synthase positive (Nisbet Brain Res Mol Brain Res 22: 329-332).

We agree with the reviewers that term “glutamatergic” is not appropriate for the entire STN as there are also GABAergic neurons in the STN. However, there is no data on exact proportions of GLUT and GABA neurons in the STN, as some markers which are clearly expressed in GABAergic neurons in cortex are not markers of GABAergic neurons in STN (e.g., presence of parvalbumin and calretinin in glutamatergic STN cells - Levesque and Parent reference).

However, we substituted “glutamatergic” to “excitatory” which is more appropriate, and conveys the correct massage - excitatory influence of STN on other structures. (see article file, r4 and r35)

r35-37: the position of the STN varies in mammals: the STN in humans is dorso-lateral to SN, dorsal of the capsula interna, lateral of the red nucleus and lateral of the lateral hypothalamic area rostral (see Mai et al, 1997, Atlas of the human brain, AP), and in the rat its position is different. Au’s could describe the human position especially in respect with part 6 (see below).

We agree with the reviewers and have made appropriate inclusion to the manuscript (article file r37-40)

r42 the cellular and cytological composition of the STN have been researched for rat, guinea-pig, cat, pig, monkey humans. It concerns: amount of cells and its cell types, types of boutons, types of vesicles, presence of interneurons (e.g. Romansky and Usunoff, 1978 Etud Balk 31:1225-1228, 1980 J Hirnforsch 21:515-521 and Anat Embryol 159: 163-180, 1983 Neurosci Lett 42:113-117, 1985 J Hirnforsch 26:259-273, 1987 J Hirnforsch 28:407-433). Cell types, also in relation to connections, have been published for humans and several other experimental animals, to name only a few: Kitai et al, 1983 J Comp Neurol 215: 245-257, rat; the Parent group e.g. Brain Res 436: 296-310 and Brain Res Bull 10: 529-537, humans, Iwahori 1978 J Comp Neurol 182: 383-397, cat.

We agree with the reviewers that this part of the manuscript is ambiguous and could mislead the reader. Therefore, we have clearly stated that there is vast data on cellular and cytological composition of the STN, while the data on the expression of the TFs in the STN is lacking. We have appropriately updated manuscript at r45 - 50 (article file).

r56-99: first some history. The neuromeric pattern has been described for the first time by Orr (1887, J Morphol 1: 311-372). W. His contributed by the description of the sulcus limitans (Hiss 1888 Abh k Sächs Ges Wiss Math-Phys Kl 14: 341-372 and in 1890 part 15: 675-735). The pro-neuromeres were already described by Berquist and Källén, see 1954 J Comp Neurol 100: 627-660 and see Tsai et al. 1981, parts I and II, J Comp Neurol 198: 275-306. By the way what is the difference between pro-neuromeres and prosomeres? It is sufficient to indicate in this part that the neuromeric pattern is unclear in pros- and mesencephalon. The conflicts in literature concern the sulcus limitans, number of neuromeres and the localization the sensory-motor columns in the prosencephalon (best citation presumably Gribnau and Geijsberts Adv Anat Embryol Cell Biol 91: 1-63). An extra problem was that the proneuromeres disappeared before the neuromeres appeared. By this time laps the two phenomena could hardly be connected. It is then the place to introduce Rubenstein’s prosomeric model.

Authors could consider to import in their article the E10.5 and/or E12.5 figure of Rubenstein published in Science 266, 1994. It makes it clear to non-hypothalamic researchers.

We thank the reviewers for this insightful comment. We are aware of the mentioned studies and their contribution to developmental neurobiology (which is often overlooked due to the age of the mentioned studies). In the original manuscript we tried to be concisely describe historical perspectives and focus on currently accepted models of hypothalamic and diencephalic development.

In revised manuscript we included the majority of proposed studies and incorporated all reviewers’ suggestions in appropriate paragraphs. Unfortunately, the extensive historical review of the development of neuromeric model, and diencephalic development in general, is out of scope of this review. We strongly believe that this topic should be discussed in a separate historical review where all necessary papers could be duly mentioned and discussed. (See the updated chapter 2-Historical perspectives)

r95-99 could be reduced Author’s should check whether all citations are needed or not.

We agree with the reviewers, thus we have reduced this section as it does not contain any essential information.

r100-116: stating that borders are present and the hypothalamus can be divided in two prosomeres, without showing the borders in an embryo makes it difficult for the reader to understand the text. This is an extra argument to import figure(s) of Rubenstein. Moreover, the text presumably can be reduced due to the extra figure(s).

As we already mentioned in reply to major issue no.3, we opted for importing the figure from Rubenstein JL, Martinez S, Shimamura K, Puelles L (1994) The embryonic vertebrate forebrain: the prosomeric model. Science 266:578-580. This figure allowed us to reduce the text regarding the hypothalamic organization and makes the details of prosomeric model more accessible to readers. (See r125 and r148 at the end of chapter 2 of the article file).

r118-175: this referee leaves to Au’s stating that the older literature had a vague definition of the subthalamic region. Nevertheless, in the whole paragraph the autoradiographic study of Keyser in the Chinese hamster is missing. This study should have been essential for Au’s. Keyser’s thesis (1972) describes the diencephalon development and in the Handbook of the hypothalamus the development of the hypothalamus has been treated and clear STN results are given:

Keyser A (1972), The development of the diencephalon of the Chinese hamster. An investigation of the validity of the criteria of subdivision of the brain. Thesis Nijmegen, The Netherlands (also publ. in Acta Anat (Basel) 83 (suppl 59: 1-178

Keyser A (1979) Development of the hypothalamus in mammals. An investigation into its morphological position during ontogenesis. In Morgan PJ, Panksepp J (eds.) Handbook of the hypothalamus vol I. Marcel Dekker Inc. NY, Basel pp65-136

This referee steals text from Marani et al., The subthalamic nucleus, Part 1. Adv Anat Embryol Cell Biol 198, to give a summary of Keyser’s work, so Au’s can decide (with excuses for typing errors in advance): The regio subthalamica in the Chinese hamster develops rostral of the synencephalon (future regio pretectalis) and basal to both the pars dorsalis (parencephalon posterior) and ventralis thalami (parencephalon anterior) (for localization of these parts on the ventricular surface see Fig. 7 ). At E 12 ( embryonic day 12) the neuromeres are clearly discernable. They show that the basal parts of the future regio pretectalis, the area below it (the future regio subthalamica), and the area caudal to the eyestalk are advanced in development compared to the other ventricular areas. At E 13 a mantle layer can be discerned in these areas. A compact “stream” (Keyser 1972) of cells can be noted and is called the subthalamic cell cord. This cell cord does not contain condensations or a development that indicates pronuclei. The cord reaches from its basal part below the future main thalamic (parencephalic) areas towards the diencephalic telencephalic border that can be recognized by the external telodiencephalic groove (also called the sulcus haemisphericus). So, early in development (E 12 -E 13 ) a subthalamic region can be discerned behind the mammillary recessus and a subthalamic cell cord extending from this area, reaching to the telencephalic-diencephalic border. At E 14 the prerubral tegmentum contains the pronuclei of the nuclei of Cajal and Darkschewitsch and it continues without clear borders in the more rostrally situated regio subthalamica. The basal area of the subthalamic cell cord is slightly more developed than the rest of this cell cord. It is this stage of development in which the growth of the mantle layer obscures the neuromeric borders. However, the neuromeric subdivisions can still be discerned in the basal/caudal diencephalic part, delineating the subthalamic area and the start of its cell cord. It should be noted here that the basal regio subthalamica is sandwiched between the tegmentum prerubralis and the regio mamillaris, while from its rostral part the subthalamic cell cord rises towards the diencephalic-telencephalic border. At E 15, within the regio subthalamica develops the supramammillary commissure that crosses the midline. The development of the regio subthalamica is advanced, and it is proposed that this area is “an organizer centre for the rostralmost parts of the brain” (Keyser 1972). The corpus subthalamicum Luysii (the future nucleus subthalamicus) is recognizable at this stage. “It develops from a tangentially migrating stream of cells that, originating from the matrix of the supramammillary recess, gradually shifts in a rostrodorsal direction” (Keyser 1972). At E 16 the subthalamic cell cord has developed into the suprapeduncular complex and a superficial corpus subthalamicum can be recognized. The suprapeduncular complex contains several subdivisions, but seemingly contributes to the entopeduncular nucleus and the globus pallidus (the rostral extension of the suprapeduncular complex). Translated into human terminology, it contributes to the globus pallidus internus and externus, respectively. A posterior hypothalamic area is considered to develop in relation to the corpus subthalamicum. The future corpus Luysii has a latero-dorsal migration, while the remnants of the subthalamic cell cord contain a more medio-dorsal development, in fact separating both cell populations. Their base is already loosened at E 16 . Thus, the subthalamic area really undergoes a tangential migration, in which the corpus Lsii takes the most lateral route. The autoradiographic results in the Chinese hamster show that the area subthalamica is characterized by an early onset of differentiation. It is autoradiographically labelled from E 12 until E 18 onwards, being one of the first labelled areas. It originates from the medially located ventricular surface of the area above the mammillary recess and spreads tangentially. The most laterally situated cells of the corpus Luysii are born on E 13 to E 18 . The tangential migration is also supported by the autoradiographic results.

Au’s can disagree with these results, but at least they should have mentioned these results, since autoradiography is a strong, basic technique in embryology and indeed can follow developing neuronal cells to a large extend.

We agree with the reviewer and accepted its suggestion. Therefore, we added appropriate text and discussed the key findings from Keyser studies. Please see r173 - 178 (article file).

r177-193: It is still worthwhile to look into the review article of Claudio Stern, who summarized in 2001 the expectations of the future research: It also seems likely that more complex mechanisms, perhaps including some degree of pre-patterning (or differential competence) of the prospective neural plate, and/or a role for differential timing of exposure to organizer-derived signals will be revealed by future research. (Stern, 2001, Stern CD (2001) Initial pattering of the central nervous system: How many organizers. Nature Reviews Neurosci 2: 92-98). Moreover, in the description of concentration gradients of secreted morphogens should be added, according to this referee: e.g. Schmidt et al. See ref in: 2008 The role of Wnt signalling in the development of somites and neural crest, in Adv Anat Embryol Cell Biol 195:1- 64. There are more researchers that contributed to where we are now (Bronner-Fraser?).

We agree with the reviewers and have added a paragraph which briefly outlines the work done by the above-mentioned authors (see revised chapter 4-Induction and early patterning of hypothalamic primordium). However, we feel that more thorough, in-depth discussion of early patterning is beyond the scope of this manuscript and would detract from the main topic.

r196-210: The underlying problem is when are meso-ectoderm cells deposited into the embryo by both the epiblast and ectoderm. See e.g. Vermeij-Keers and Poelmann, 1980 Neth J Zool 30: 74-81; Smits van Prooije et al., 1985, Anat Embryol 176: 71-77. A better worked out text could be made.

We agree with the reviewers that this is a very important topic. The suggested references and the work the authors have done are crucial for elucidating mechanisms of neural crest formation, however, we feel that an in-depth discussion of these works is beyond the scope of our review, since it focuses on hypothalamic development and subthalamic nucleus.

Nevertheless, we have briefly outlined different theories of floor plate induction, especially of hypothalamic floor plate induction.

r195-270: SHH and Wnt do appear in various segments/ neuromeres/meso-ectoderm cells. The appearance of both morphogens don’t solely characterise distinct neuronal localisations (pure topography), but is also the consequences of general functional processes belonging to development and these processes are subject to time lapses within one and the same area. In other words, using these morphogens as being exclusively a local marker seems unwarranted, since these morphogens pass over neurons and neuronal networks in a wave-like manner or can be considered as an autonomous progression of differentiation. (see Chandebois and Faber, Automation in animal development, Monographs in Developmental Biology ed. Wolsky A, 16: 1-204). No argument can be borrowed from these morphogens to prove that the STN is hypothalamic (hp1 or hp2) in origin.

We thank the reviewers on the insightful comment. Although, we do not agree completely with the statement made by the reviewers, we feel it is important for the readers to be exposed to all opposing views. Therefore, we added a short paragraph dealing with proposed theories (see r264 - 271).

r293-296: Fg/8 is only postulated to play an important role in STN development, because Fg/8 is involved in the development of the retromammilary area(?): Clearly a postulate.

Parts 4.1 to 4.3 are weak arguments to place these morphogens in a clear relation to early STN-hypothalamic development.

We agree with the reviewers that morphogens appear in various segments of the neural tube. However, if a morphogen is important for the specification of a site of origin of the STN (hypothalamic primordium), we thought it was important to discuss it. Furthermore, the intention of this review was not to provide a definitive answer to which morphogens and TFs are crucial for STN development, but to summarize all available data linked to the STN development and to stimulate future research on this topic.

Nevertheless, we expanded these paragraphs to emphasize that these morphogens influence the patterning and the morphogenesis of the whole neural tube. (See revised chapters on Shh, Wnts and Fgfs)

r311-463 (5.1) contains a fine description of the transcription factors present in neural progenitors: remark on Dbx1, the arcuate nucleus contains dopaminergic cells and on Nkx2-1, the tangential movement has been described by Keyser for the first time.

We thank the reviewer for the comment on transcription factors. Regarding the Dbx1 and arcuate nucleus, we are well aware that arcuate nucleus contains various neuropeptides (see Chronwall, B. M. (1985). Anatomy and physiology of the neuroendocrine arcuate nucleus. Peptides, 6, 1-11; Chan-Palay, V. et al (1984). Distribution of tyrosine-hydroxylase-immunoreactive neurons in the hypothalamus of rats. J. Comp. Neurol. 227, 467-496). However, studies presented in the manuscript provide data on the appearance of orexigenic neurons specified by Dbx1 (and in that particular case dopaminergic neurons were not affected by the loss of Dbx1). We also appreciate the notion that STN’s neurons migrate tangentially, which we discussed when describing Keyser’s work (see chapter 3)

r465-566 (5.2) good overview of the transcription factors in postmitotic neurons

We thank the reviewers for the comment.

r611-685 (6) On first reading this referee thought to skip part 6 till he realised that it is the construct of the text. Proposal: 1) show that deficiencies in morphogens or transcription factors lead to aberrations or deflections in organs or tissues (back ground), e.g. like in Clinical neuroembryology (Springer) by ten Donkelaar, Lammens and Hori. I suppose that the Au’s do have more and presumably better examples (plural!). 2) Take one of the disorder examples that have been well worked out (convincing argument) 3) summarize the other cases. It is a temptation to relate the early developmental morphogens and transcription factors to neurodevelopmental disorders and therefore the description should stay in.

We agree with the reviewers that this section could be more focused and clearer. We have shortened it by removing some non-essential parts and added new text according to reviewers’ suggestions. As we mentioned in the text, the purpose of this paragraph is to draw attention to the STN development and the fact it has much broader role than just motor control. We agree with the reviewers that there is no evidence of STN involvement in the pathophysiology of NDDs (as we now clearly state in the manuscript) and that some connections that we made can have different interpretations. However, we feel that evident lack of data on human STN development needs to be corrected and that one of the best ways to do it is to study NDDs. We are also aware that some of the statements made are at best speculative, but their purpose is to provoke the readers to possibly address these questions in their future work.

Small errors or indistinctions that should be corrected:

r576: Marani, Subthalamic nucleus part 1 does not say that the STN is homogeneous. Look into part 2 (Heida, The subthalamic nucleus part II Modelling and simulation activity, Springer, 2008) at the end you will find the tuning of part 1 and 2 in which based on physiology two types of neurons are discerned and the presence of interneurons is described.

We are aware of the cellular composition of the STN, and agree with the reviewers that this statement could be misleading (as it was not intended to describe neuronal populations). Therefore, we have removed this statement and adjusted the manuscript accordingly (see r685-687).

r581-583: “” there is a set of shared TFs 582 between the STN, mammillary, supramammillary, and ventral premammillary nuclei, and mesodiencephalic dopaminergic neurons"”, but what then determines the STN?

We agree with the reviewers that this is a “million dollar question”. As mentioned throughout the manuscript, the specification of the STN is not due to the single gene, but rather a transcriptional network in which many individual TF are also expressed in other transcriptional networks specifying other structures. The crucial thing is most likely the timing of TFs’ expression combined with its location and leve of expressionl. Therefore, we currently cannot specify what is crucial for the specification of the STN but rather which “players” are involved (see r690-693).

In line with the above mentioned statements we have added short text to the manuscript.

r587-588: Dbx1 presumably gives rise to glutamatergic populations. Fact or presupposition?

According to the study by Nouri and Awatramani (2017) Dbx1 is expressed in the region where future glutamatergic nuclei are generated (e.g.STN, PSTN, supramammillary nucleus). It is impossible, at this moment, to state if Dbx1 is important for the glutamatergic specification in any way. However, we felt that this notion should be mentioned so that readers be aware of the possibility. We feel that additional studies are needed to elucidate this question (as authors themselves stated).
